# Supplementary material for: Phylogenetic Analysis with Prediction of Cofactor or Ligand Binding for Pseudomonas aeruginosa PAS and Cache Domains
Source: Microbiol Spectr. 2021 Dec 22;9(3):e01026-21. doi: 10.1128/spectrum.01026-21 (PMC8694187; doi:10.1128/spectrum.01026-21)
Supplement: SUPPLEMENTAL FILE 1 — Supplemental material. Download SPECTRUM01026-21_Supp_1_seq3.pdf, PDF file, 7.4 MB [file spectrum01026-21_supp_1_seq3.pdf]

Supplement to:

Phylogenetic analysis with prediction of cofactor or ligand binding for *Pseudomonas aeruginosa* PAS and Cache domains

Andrew Hutchin[a,b,c,d*], Charlotte Cordery[a,b,c], Martin A. Walsh[b,c], Jeremy S. Webb[a,e] and Ivo Tews[a,e,#]

[a] Biological Sciences, Institute for Life Sciences, University of Southampton, UK

[b] Diamond Light Source, Harwell Science and Innovation Campus, Didcot OX11 0DE UK

[c] Research Complex at Harwell, Harwell Science and Innovation Campus, Didcot OX11 0FA, UK

[d] Structure and Function of Biological Membranes Lab, Université Libre de Bruxelles, Belgium

[e] National Biofilms Innovation Centre, University of Southampton, UK

Running Head: PAS domains in *Pseudomonas aeruginosa* PAO1 proteins

# Address correspondence to Ivo Tews, ivo.tews@soton.ac.uk

[*] Present Address: Evotec (UK) Ltd, Abingdon, UK

To establish the phylogeny of PAS and Cache domains we show a maximum likelihood tree as Figure 1 in this paper. We sought to compare this tree to phylogenetic trees constructed using different models, namely neighbor-joining [1]; maximum parsimony with a Subtree-Pruning-Regrafting algorithm [2]; minimum evolution with an initial tree generated using a neighbor-joining algorithm [1, 3], and searched using the Close-Neighbor-Interchange algorithm [2]; and an unweighted pair group method with arithmetic mean (UPGMA) [4]. In all cases and identical to the analysis presented in the main paper, sequences were aligned using CLUSTALW, as implemented in MEGA7 [5, 6], before subsequent tree generation using MEGA7. The reader may evaluate these trees for differences, and we note some hallmarks of a comparison here:

Within the **neighbor-joining tree**, clades with similar ligand or cofactor binding formed for reference PAS and Cache domains occur in a smaller number of bootstrap replicates than within the maximum-likelihood tree. For example, a clade containing all FMN-binding PAS domains from the reference dataset is formed in 78 bootstrap replicates when analyzed using maximum-likelihood, but the equivalent clade is only formed in 21 bootstrap replicates when analyzed with neighbor-joining. As a result of this difference, we have included all clades formed in more than 10 bootstrap replicates of the neighbor-joining tree for further analysis, as this provides largely unambiguous clustering of the reference PAS and Cache domains. When the neighbor-joining tree is analyzed using this cut-off, the majority of the clades formed between the PAO1 PAS or Cache domains and reference domains are similar to the ones identified in the maximum-likelihood tree. The exceptions to this are the clade formed between PA5381 PAS1 and the heme-b binding, enzymatic, PAS domain of *Pm*HodM and the clade formed between PA1347 PAS1 and the auto-inducer binding PAS domain of *Vc*VqmA. However, a number of clades are formed in the maximum-likelihood tree that are not identified through neighbor-joining, suggesting that when used in this way, maximum-likelihood is the more sensitive method for identifying phylogenetic relationships between PAS and Cache domains.

It is immediately seen from the **maximum parsimony tree** that FMN binders are split into several, smaller clades, and several clades are also observed for the non-ligand / cofactor binding group. It is worth noting that the clades for amino acid binding and carboxylic acid binding domains are similar to those within the tree constructed with the maximum likelihood method. However, in contrast to the maximum likelihood tree, several small clades or pairings of PA

domains are formed without reference, e.g. the five PAS domains PA5017 PAS2, PA5442 PAS2, PA4601 PAS4, PA0285 PAS2 and PA0575 PAS4. Further different observations to the maximum likelihood tree include the grouping of PA0172 dCache with the cytokinin binding PAS domain of *At*AHK4, PA5361 PAS1 with the non-ligand/cofactor binding PAS domain of *Ec*DhR, PA1098 PAS1 with the FMN binding PAS domain of EI222 PAS, PA0464 sCache with the amino acid binding dCache domain of *Cj*Tlp3, and PA2870 PAS1 with the metal binding PAS domain of *Pa*CzcS. These differences might be explored with experimental validation.

The **minimum evolution tree** shows amino acid binding and carboxylic acid binding clades similar to the maximum likelihood tree. However, similar to the maximum parsimony tree the tree fails to cluster FMN binders, and it also groups PA0172 dCache with the cytokinin binding PAS domain of *At*AHK4, which may give further support to this observation. Another difference to the maximum likelihood three is a clade that is identified for the PAS domains PA0285 PAS1, PA0338 PAS1, PA5442 PAS1, PA1181 PAS2 and PA4112 PAS3; these sequences grouped with non-ligand / cofactor group in the maximum likelihood tree. Non-ligand / cofactor binders are divided across several clades within the minimum evolution tree, potentially providing additional insight into relationship of these domains.

Finally, the **UPGMA** method (unweighted pair group method with arithmetic mean) adds many small clades of PA domains to the analysis. An example is PA1347 PAS1 that is grouped with PA1261 PAS1 and the auto-inducer binding PAS domain of *Vc*VqmA, which is similar to observation made with the neighbor-joining tree. The amino acid binding and the carboxylic acid binding clades are similar to the maximum likelihood analysis. The PA0172 dCache is grouped with the cytokinin binding PAS domain of *At*AHK4, similar to maximum parsimony and minimum evolution trees; hence, comparing all trees this clade is the only one not covered within the maximum likelihood tree. The UPGMA tree may have the advantage that additional PA domains being identified as grouped with the FAD and FMN binders.

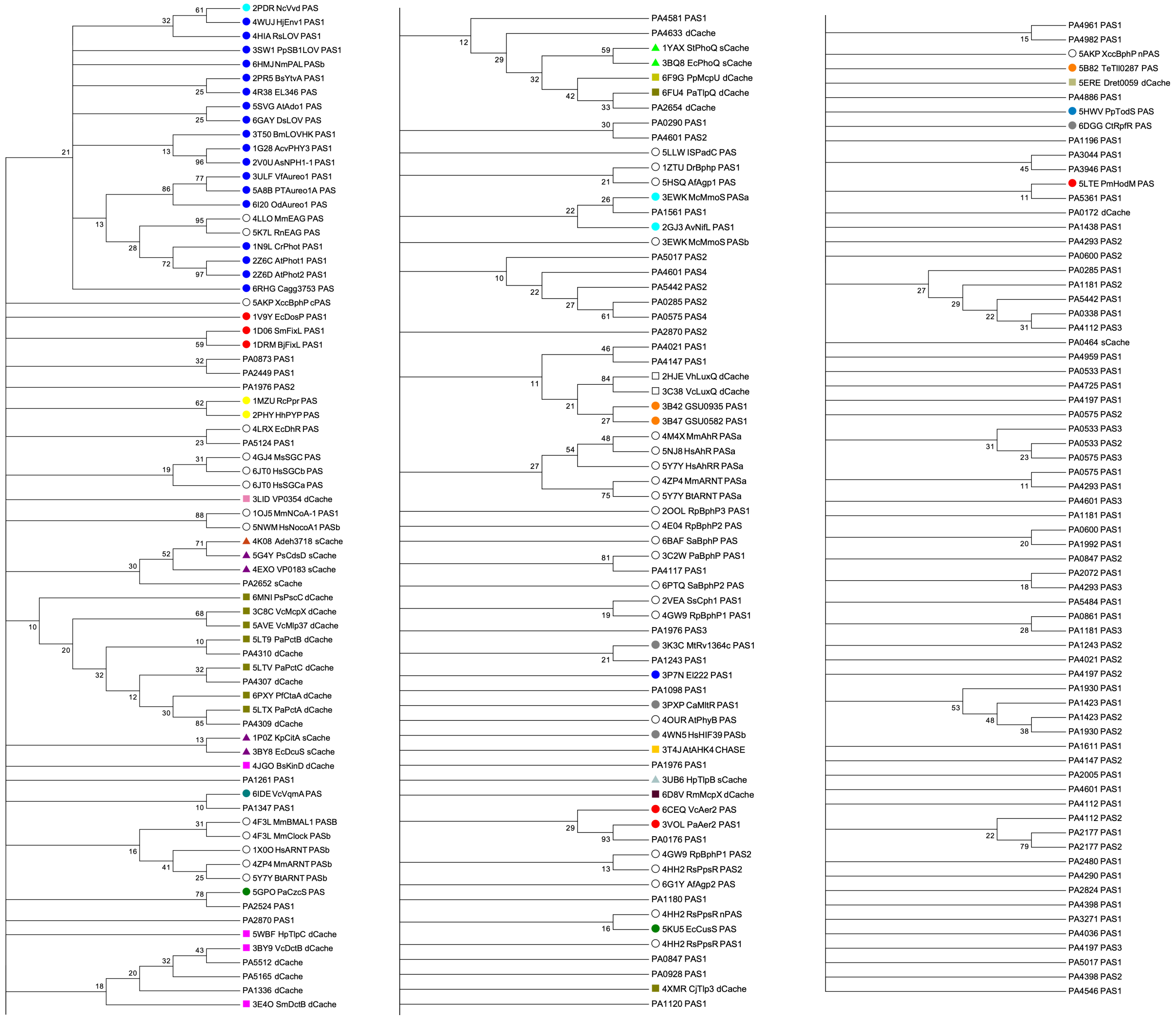

Neighbor-joining phylogenetic tree [1], labelled similarly to Figure 1. The percentage of bootstrap replicates that reproduced each branch is given, with branches corresponding to less than 10% bootstrap replicates collapsed for clarity.

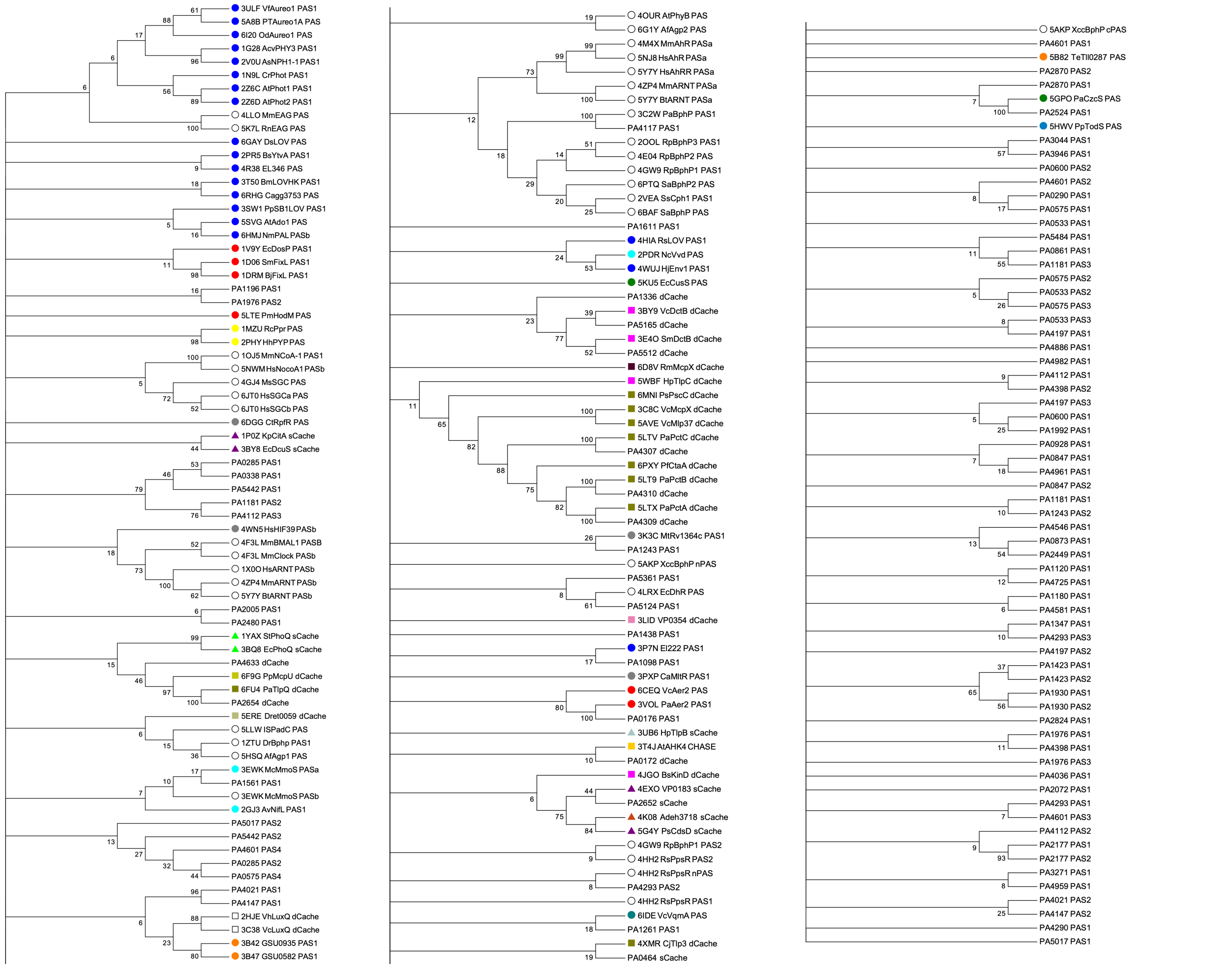

Maximum parsimony phylogenetic tree with a Subtree-Pruning-Regrafting algorithm [2], labelled similarly to Figure 1. The percentage of bootstrap replicates that reproduced each branch is given, with branches corresponding to less than 5% bootstrap replicates collapsed for clarity.

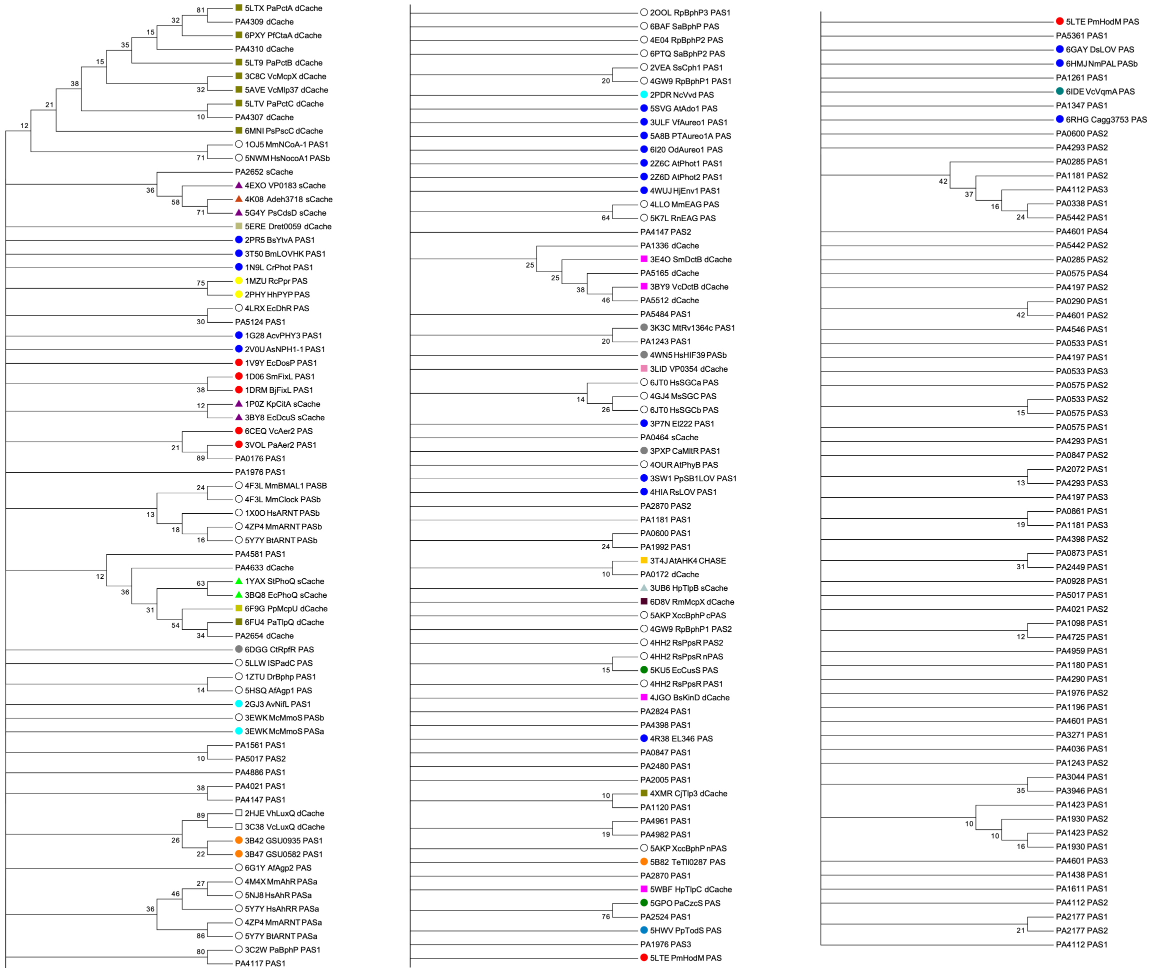

Minimum evolution phylogenetic tree with an initial tree generated using a neighbor-joining algorithm [1, 3], labelled similarly to Figure 1. The percentage of bootstrap replicates that reproduced each branch is given, with branches corresponding to less than 10% bootstrap replicates collapsed for clarity.

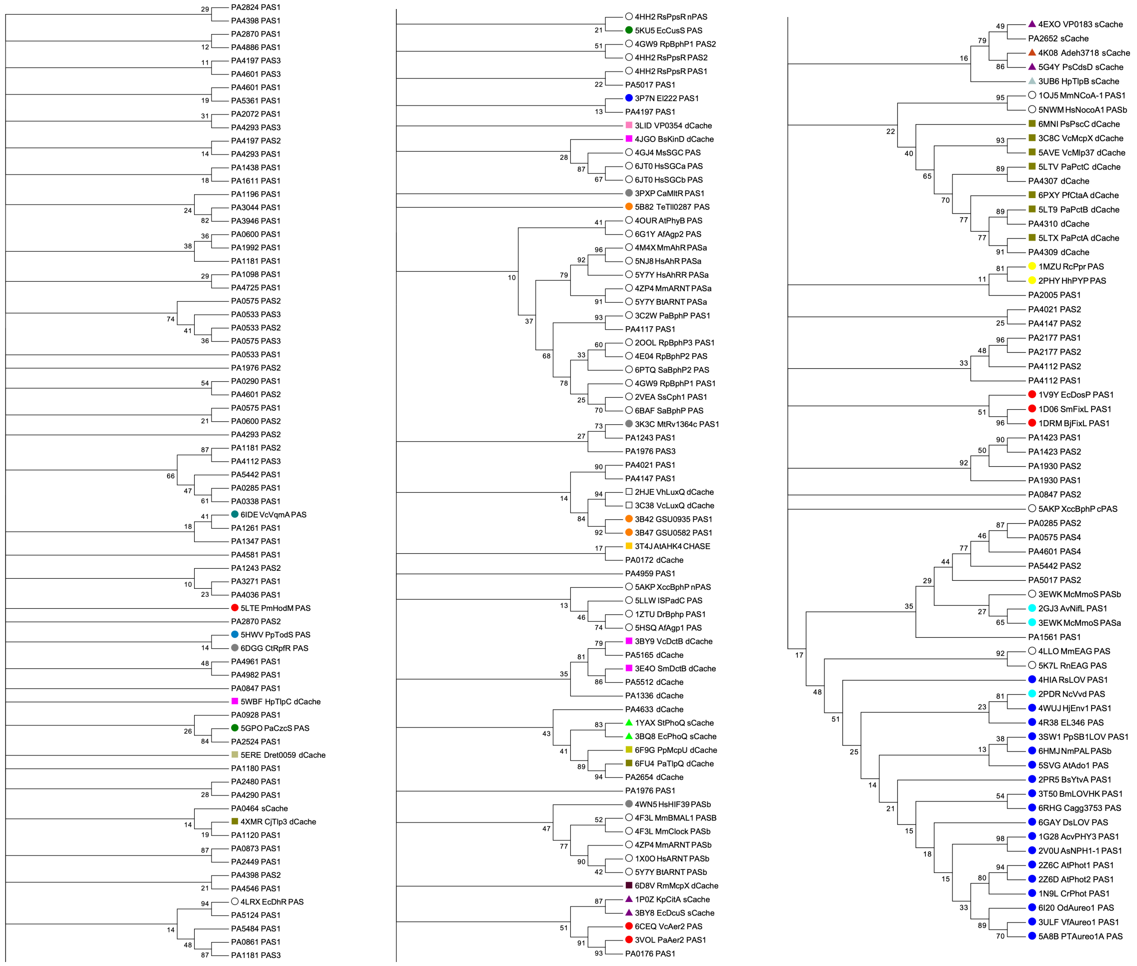

UPGMA phylogenetic tree constructed with a Close-Neighbor-Interchange algorithm [2] and an unweighted pair group method with arithmetic mean [4], labelled similarly to Figure 1. The percentage of bootstrap replicates that reproduced each branch is given, with branches corresponding to less than 10% bootstrap replicates collapsed for clarity.

References:

1. Saitou, N. and M. Nei, Mol Biol Evol, 1987. **4**(4): p. 406-25.
2. Nei, M. and S. Kumar, *Molecular evolution and phylogenetics*. 2000, Oxford ; New York: Oxford University Press. xiv, 333 p.
3. Rzhetsky, A. and M. Nei, Molecular Biology and Evolution, 1992. **9**(5): p. 945-967.
4. Sneath, P.H.A. and R.R. Sokal, *Numerical taxonomy; the principles and practice of numerical classification*. A Series of books in biology. 1973, San Francisco,: W. H. Freeman. xv, 573 p.
5. Thompson, J.D., D.G. Higgins, and T.J. Gibson, Nucleic acids research, 1994. **22**(22): p. 4673-4680.
6. Kumar, S., G. Stecher, and K. Tamura, Molecular biology & evolution, 2016. **33**(7): p. 1870-1874.
7. Larkin, M.A., G. Blackshields, N.P. Brown, R. Chenna, P.A. McGettigan, H. McWilliam, F. Valentin, I.M. Wallace, A. Wilm, R. Lopez, J.D. Thompson, T.J. Gibson, and D.G. Higgins, Bioinformatics, 2007. **23**(21): p. 2947-8.
8. Drozdetskiy, A., C. Cole, J. Procter, and G.J. Barton, Nucleic Acids Res, 2015. **43**(W1): p. W389-94.

The following pages show sequence alignments of clusters shown in Fig. 1, some of which were discussed in detail in the text. Sequences were aligned using CLUSTALW, as implemented in MEGA7 [5, 6], and displayed using the CLUSTALX graphical interface [7]. Secondary structure is indicated in red for alpha helices and in green for beta strands, as assigned from the following structures: 6RHG for FMN, 2GJ3 for FAD, 2PHY for HA, 3BY9 for dCache carboxylic acids, 3BY8 for sCache carboxylic acids, 4XMR for amino acids, 1YAX for sCache Metals, 4E04 for non-ligand group, arrow 2, 4WN5 for fatty acids, 1D06 for Heme b, 6IDE for autoinducers, 3T4J for cytokinins, 5GPO for PAS metals. The alignment branch marked with arrow 3 showing four PAS sequences was submitted to JPRED4 [8] and the predicted secondary structure was used for annotation.

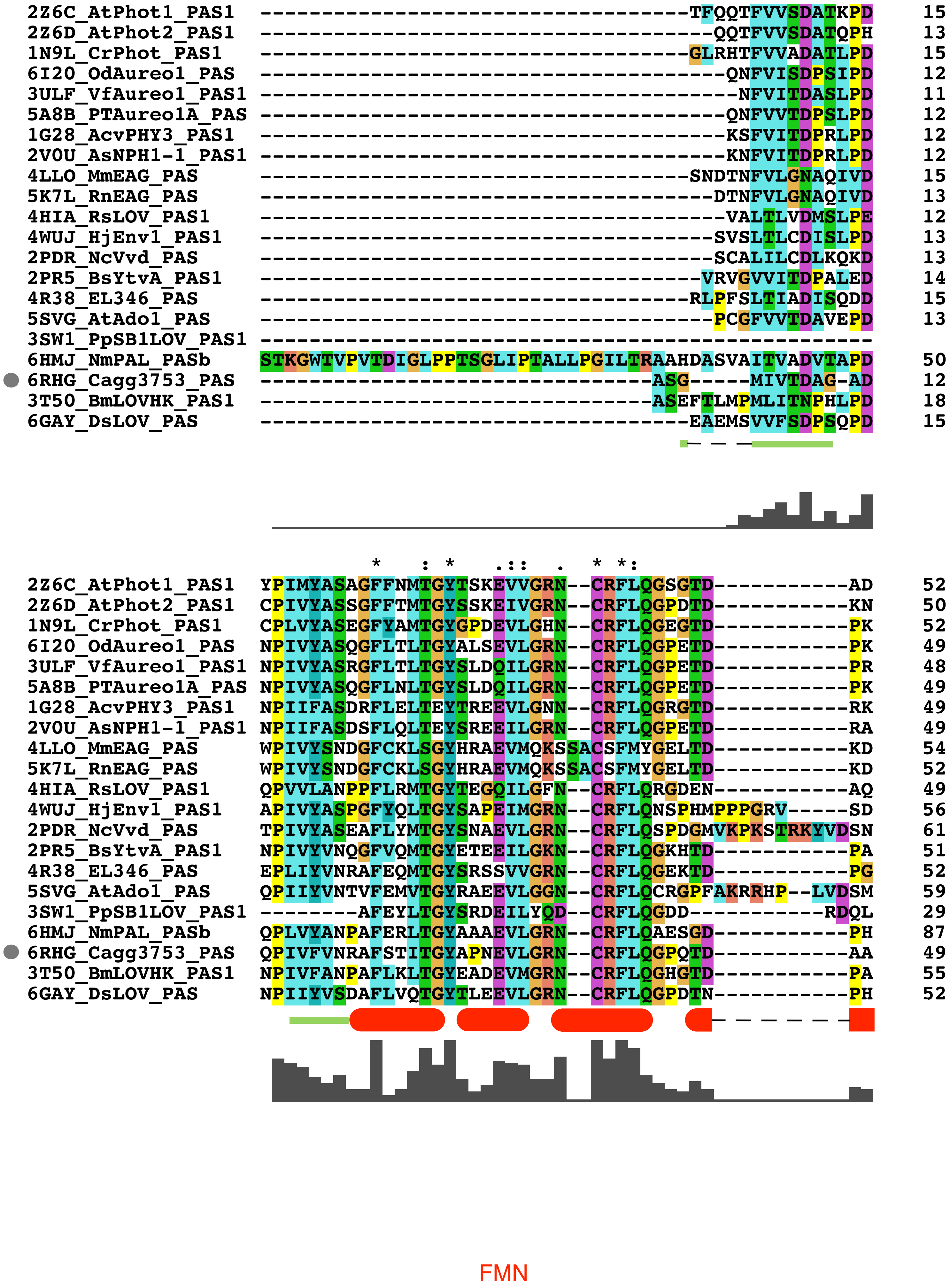
2Z6C_AtPhot1_PAS1 ------------------------------------TFQQTFVVSDATKPD 15
2Z6D_AtPhot2_PAS1 --------------------------------------QQTFVVSDATQPH 13
1N9L_CrPhot_PAS1 ------------------------------------GLRHTFVVADATLPD 15
6I20_OdAureo1_PAS ---------------------------------------QNFVISDPSIPD 12
3ULF_VfAureo1_PAS1 --NFVITDASLPD 11
5A8B_PTAureo1A_PAS ---------------------------------------QNFVVTDPSLPD 12
1G28_AcvPHY3_PAS1 ---------------------------------------KSFVITDPRLPD 12
2V0U_AsNPH1-1_PAS1 ---------------------------------------KNFVITDPRLPD 12
4LLO_MmEAG_PAS ------------------------------------SNDTNFVLGNAQIVD 15
5K7L_RnEAG_PAS --------------------------------------DTNFVLGNAQIVD 13
4HIA_RsLOV_PAS1 ---------------------------------------VALTLVDMSLPE 12
4WUJ_HjEnv1_PAS1 --------------------------------------SVSLTLCDISLPD 13
2PDR_NcVvd_PAS --------------------------------------SCALILCDLKQKD 13
2PR5_BsYtvA_PAS1 -------------------------------------VRVGVVITDPALED 14
4R38_EL346_PAS ------------------------------------RLPFSLTIADISQDD 15
5SVG_AtAdo1_PAS --------------------------------------PCGFVVTDAVEPD 13
3SW1_PpSB1LOV_PAS1 ---
6HMJ_NmPAL_PASb STKGWTVPVTDIGLPPTSGLIPTALLPGILTRAAHDASVAITVADVTAPD 50
6RHG_Cagg3753_PAS ---------------------------------ASG-----MIVTDAG-AD 12
3T50_BmLOVHK_PAS1 ---------------------------------ASEFTLMPMLITNPHLPD 18
6GAY_DsLOV_PAS ------------------------------------EAEMSVVFSDPSQPD 15
* : * .:: . * *:
2Z6C_AtPhot1_PAS1 YPIMYASAGFFNMTGYTSKEVVGRN--CRFLQGSGTD-----------AD 52
2Z6D_AtPhot2_PAS1 CPIVYASSGFFTMTGYSSKEIVGRN--CRFLQGPDTD-----------KN 50
1N9L_CrPhot_PAS1 CPLVYASEGFYAMTGYGPDEVLGHN--CRFLQGEGTD-----------PK 52
6I20_OdAureo1_PAS NPIVYASQGFLTLTGYALSEVLGRN--CRFLQGPETD-----------PK 49
3ULF_VfAureo1_PAS1 NPIVYASRGFLTLTGYSLDQILGRN--CRFLQGPETD-----------PR 48
5A8B_PTAureo1A_PAS NPIVYASQGFLNLTGYSLDQILGRN--CRFLQGPETD-----------PK 49
1G28_AcvPHY3_PAS1 NPIIFASDRFLELTEYTREEVLGNN--CRFLQGRGTD-----------RK 49
2V0U_AsNPH1-1_PAS1 NPIIFASDSFLQLTEYSREEILGRN--CRFLQGPETD-----------RA 49
4LLO_MmEAG_PAS WPIVYSNDGFCKLSGYHRAEVMQKSSACSFMYGELTD-----------KD 54
5K7L_RnEAG_PAS WPIVYSNDGFCKLSGYHRAEVMQKSSACSFMYGELTD-----------KD 52
4HIA_RsLOV_PAS1 QPVVLANPPFLRMTGYTEGQILGFN--CRFLQRGDEN-----------AQ 49
4WUJ_HjEnv1_PAS1 APIVYASPGFYQLTGYSAPEIMGRN--CRFLQNSPHMPPPGRV-----SD 56
2PDR_NcVvd_PAS TPIVYASEAFLYMTGYSNAEVLGRN--CRFLQSPDGMVKPKSTRKYVDSN 61
2PR5_BsYtvA_PAS1 NPIVYVNQGFVQMTGYETEEILGKN--CRFLQGKHTD-----------PA 51
4R38_EL346_PAS EPLIYVNRAFEQMTGYSRSSVVGRN--CRFLQGEKTD-----------PG 52
5SVG_AtAdo1_PAS QPIIYVNTVFEMVTGYRAEEVLGGN--CRFLQCRGPFAKRRHP--LVDSM 59
3SW1_PpSB1LOV_PAS1 --------AFEYLTGYSRDEILYQD--CRFLQGDD-----------RDQL 29
6HMJ_NmPAL_PASb QPLVYANPAFERLTGYAAAEVLGRN--CRFLQAESGD-----------PH 87
6RHG_Cagg3753_PAS QPIVFVNRAFSTITGYAPNEVLGRN--CRFLQGPQTD-----------AA 49
3T50_BmLOVHK_PAS1 NPIVFANPAFLKLTGYEADEVMGRN--CRFLQGHGTD-----------PA 55
6GAY_DsLOV_PAS NPIIYVSDAFLVQTGYTLEEVLGRN--CRFLQGPDTN-----------PH 52
FMN

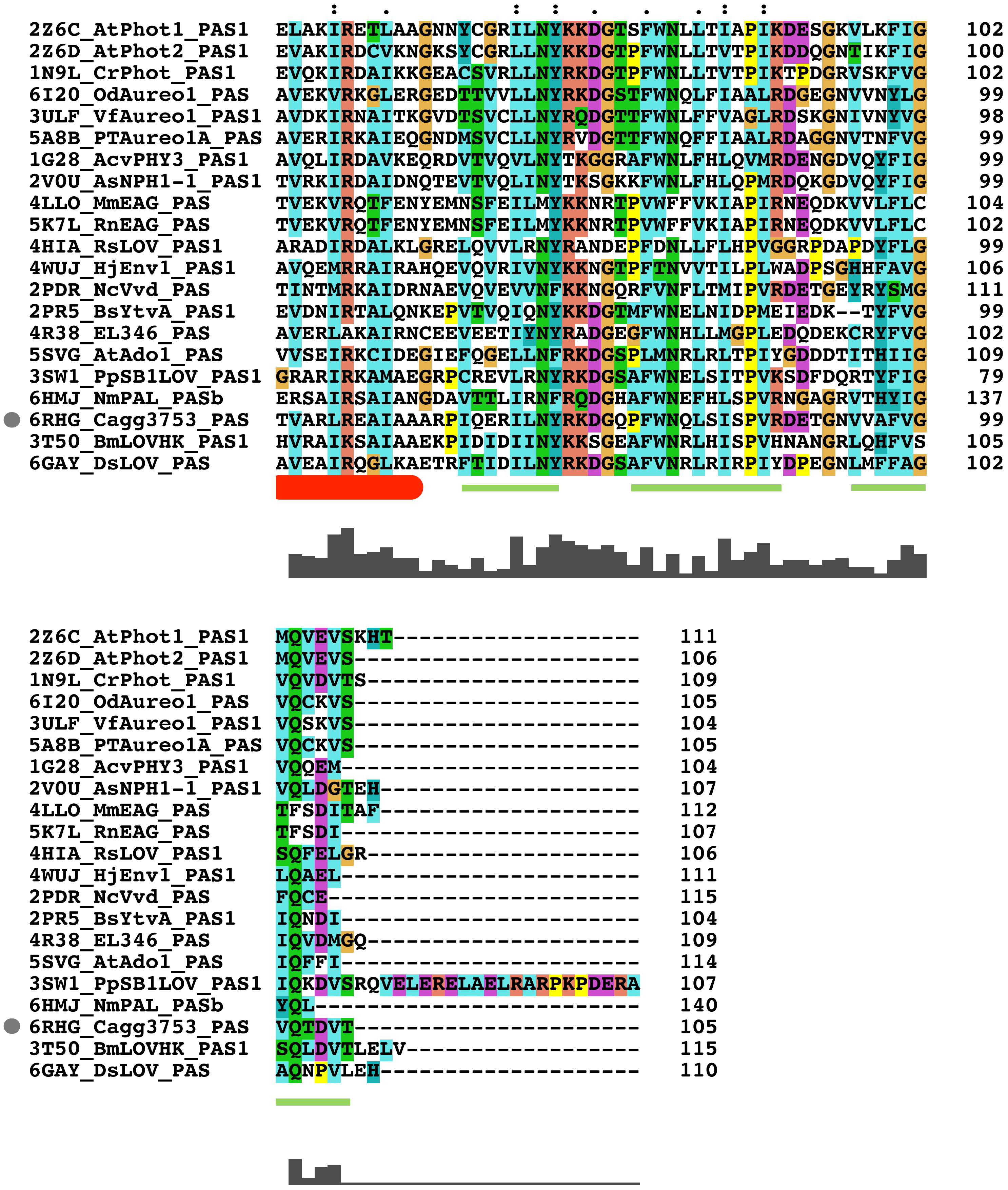
: . : : . . . : :
2Z6C_AtPhot1_PAS1 ELAKIRETLAAGNNYCGRILNYKKDGTSFWNLLTIAPIKDESGKVLKFIG 102
2Z6D_AtPhot2_PAS1 EVAKIRDCVKNGKSYCGRLLNYKKDGTPFWNLLTVTPIKDDQGNTIKFIG 100
1N9L_CrPhot_PAS1 EVQKIRDAIKKGEACSVRLLNYRKDGTPFWNLLTVTPIKTPDGRVSKFVG 102
6I20_OdAureo1_PAS AVEKVRKGLERGEDTTVVLLNYRKDGSTFWNQLFIAALRDGEGNVVNYLG 99
3ULF_VfAureo1_PAS1 AVDKIRNAITKGVDTSVCLLNYRQDGTTFWNLFFVAGLRDSKGNIVNYVG 98
5A8B_PTAureo1A_PAS AVERIRKAIEQGNDMSVCLLNYRVDGTTFWNQFFIAALRDAGGNVTNFVG 99
1G28_AcvPHY3_PAS1 AVQLIRDAVKEQRDVTVQVLNYTKGGRAFWNLFHLQVMRDENGDVQYFIG 99
2V0U_AsNPH1-1_PAS1 TVRKIRDAIDNQTEVTVQLINYTKSGKKFWNLFHLQPMRDQKGDVQYFIG 99
4LLO_MmEAG_PAS TVEKVRQTFENYEMNSFEILMYKKNRTPVWFFVKIAPIRNEQDKVVLFLC 104
5K7L_RnEAG_PAS TVEKVRQTFENYEMNSFEILMYKKNRTPVWFFVKIAPIRNEQDKVVLFLC 102
4HIA_RsLOV_PAS1 ARADIRDALKLGRELQVVLRNYRANDEPFDNLLFLHPVGGRPDAPDYFLG 99
4WUJ_HjEnv1_PAS1 AVQEMRRAIRAHQEVQVRIVNYKKNGTPFTNVVTILPLWADPSGHHFAVG 106
2PDR_NcVvd_PAS TINTMRKAIDRNAEVQVEVVNFKKNGQRFVNFLTMIPVRDETGEYRYSMG 111
2PR5_BsYtvA_PAS1 EVDNIRTALQNKEPVTVQIQNYKKDGTMFWNELNIDPMEIEDK--TYFVG 99
4R38_EL346_PAS AVERLAKAIRNCEEVEETIYNYRADGEGFWNHLLMGPLEDQDEKCRYFVG 102
5SVG_AtAdo1_PAS VVSEIRKCIDEGIEFQGELLNFRKDGSPLMNRLRLTPIYGDDDTITHIIG 109
3SW1_PpSB1LOV_PAS1 GRARIRKAMAEGRPCREVLRNYRKDGSAFWNELSITPVKSDFDQRTYFIG 79
6HMJ_NmPAL_PASb ERSAIRSAIANGDAVTTLIRNFRQDGHAFWNEFHLSPVRNGAGRVTHYIG 137
6RHG_Cagg3753_PAS TVARLREAIAAARPIQERILNYRKDGQPFWNQLSISPVRDETGNVVAFVG 99
3T50_BmLOVHK_PAS1 HVRAIKSAIAAEKPIDIDIINYKKSGEAFWNRLHISPVHNANGRLQHFVS 105
6GAY_DsLOV_PAS AVEAIRQGLKAETRFTIDILNYRKDGSAFVNRLRIRPIYDPEGNLMFFAG 102
2Z6C_AtPhot1_PAS1 MQVEVSKHT------------------- 111
2Z6D_AtPhot2_PAS1 MQVEVS---------------------- 106
1N9L_CrPhot_PAS1 VQVDVTS--------------------- 109
6I20_OdAureo1_PAS VQCKVS---------------------- 105
3ULF_VfAureo1_PAS1 VQSKVS---------------------- 104
5A8B_PTAureo1A_PAS VQCKVS---------------------- 105
1G28_AcvPHY3_PAS1 VQQEM----------------------- 104
2V0U_AsNPH1-1_PAS1 VQLDGTEH-------------------- 107
4LLO_MmEAG_PAS TFSDITAF-------------------- 112
5K7L_RnEAG_PAS TFSDI----------------------- 107
4HIA_RsLOV_PAS1 SQFELGR--------------------- 106
4WUJ_HjEnv1_PAS1 LQAEL----------------------- 111
2PDR_NcVvd_PAS FQCE------------------------ 115
2PR5_BsYtvA_PAS1 IQNDI----------------------- 104
4R38_EL346_PAS IQVDMGQ--------------------- 109
5SVG_AtAdo1_PAS IQFFI----------------------- 114
3SW1_PpSB1LOV_PAS1 IQKDVSRQVELERELAELRARPKPDERA 107
6HMJ_NmPAL_PASb YQL------------------------- 140
6RHG_Cagg3753_PAS VQTDVT---------------------- 105
3T50_BmLOVHK_PAS1 SQLDVTLELV------------------ 115
6GAY_DsLOV_PAS AQNPVLEH-------------------- 110

FMN

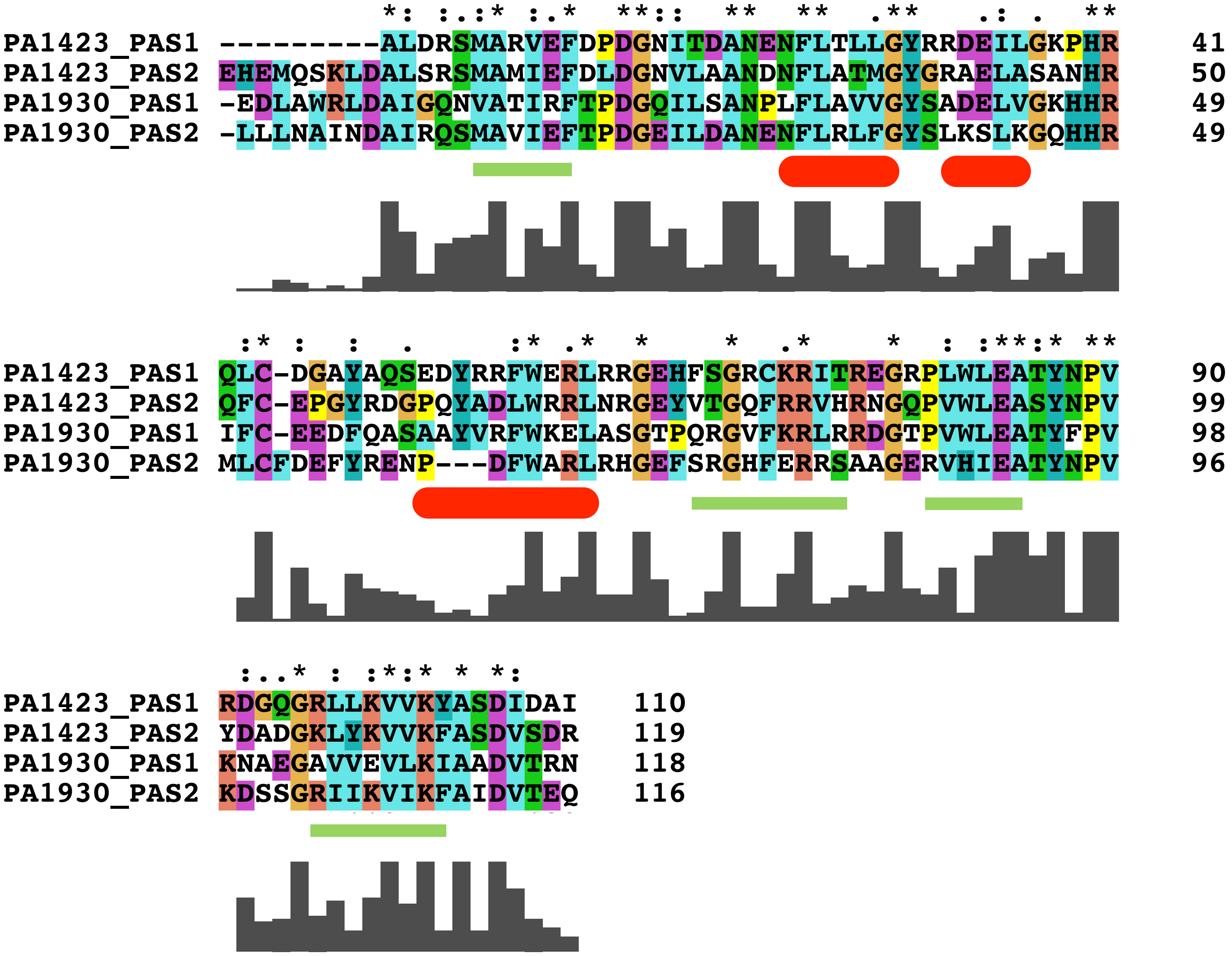
: :.: :.* **:: ** ** .** .: . **
PA1423_PAS1 ---------ALDRSMARVEFDPDGNITDANENFLTLLGYRRDEILGKPHR 41
PA1423_PAS2 EHEMQSKLDALSRSMAMIEFDLDGNVLAANDNFLATMGYGRAELASANHR 50
PA1930_PAS1 -EDLAWRLDAIGQNVATIRFTPDGQILSANPLFLAVVGYSADELVGKHHR 49
PA1930_PAS2 -LLLNAINDAIRQSMAVIEFTPDGEILDANENFLRLFGYSLKSLKGQHHR 49
:* : : . :* .* * * .* * : :**:* **
PA1423_PAS1 QLC-DGAYAQSEDYRRFWERLRRGEHFSGRCKRITREGRPLWLEATYNPV 90
PA1423_PAS2 QFC-EPGYRDGPQYADLWRRLNRGEYVTGQFRRVHRNGQPVWLEASYNPV 99
PA1930_PAS1 IFC-EEDFQASAAYVRFWKELASGTPQRGVFKRLRRDGTPVWLEATYFPV 98
PA1930_PAS2 MLCFDEFYRENP---DFWARLRHGEFSRGHFERRSAAGERVHIEATYNPV 96
:..* : :*:* * *:
PA1423_PAS1 RDGQGRLLKVVKYASDIDAI 110
PA1423_PAS2 YDADGKLYKVVKFASDVSDR 119
PA1930_PAS1 KNAEGAVVEVLKIAADVTRN 118
PA1930_PAS2 KDSSGRIIKVIKFAIDVTEQ 116

Arrow 3

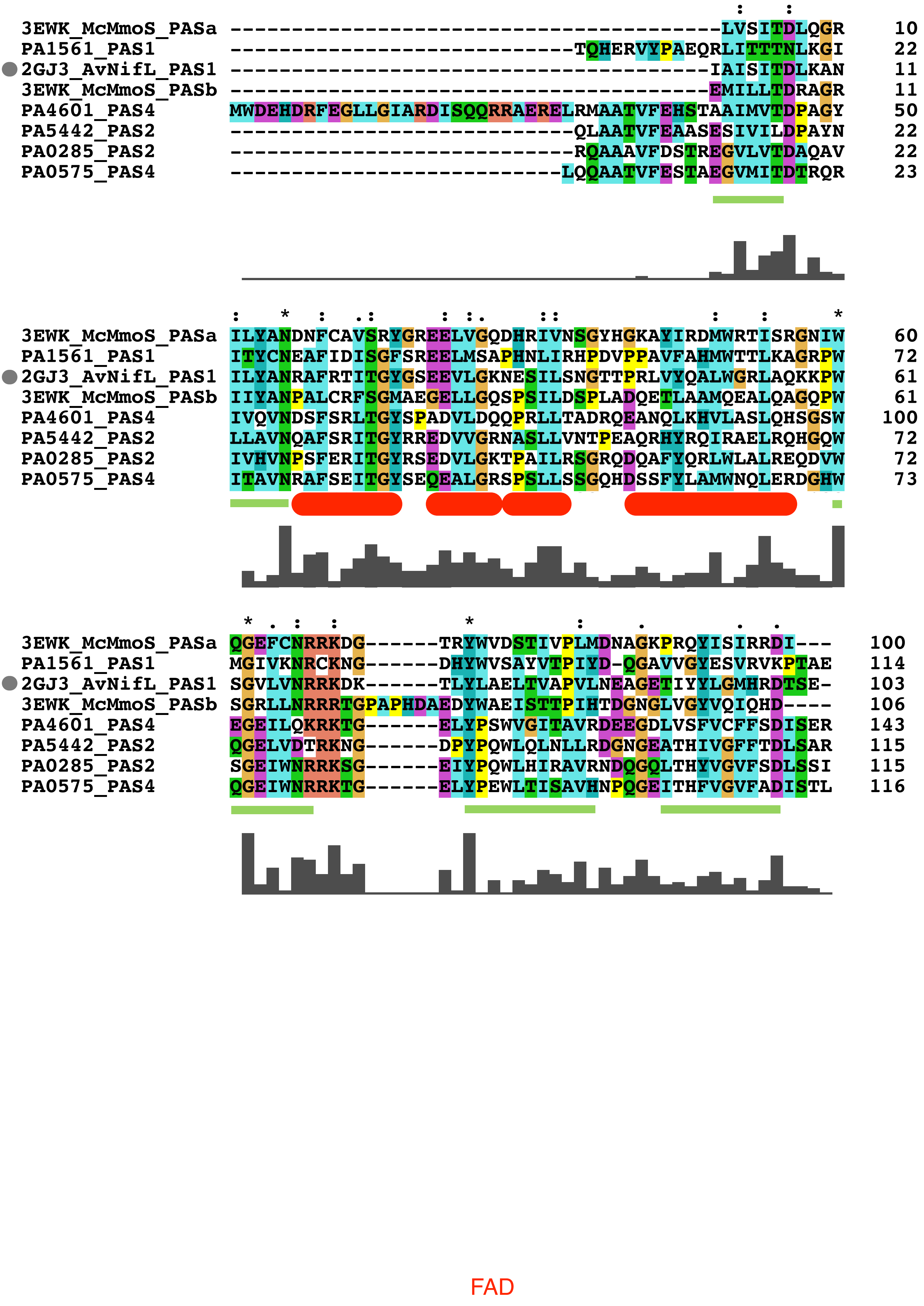

: :
3EWK_McMmoS_PASa --LVSITDLQGR 10
PA1561_PAS1 ----------------------------TQHERVYPAEQRLITTTNLKGI 22
2GJ3_AvNifL_PAS1 ---------------------------------------IAISITDLKAN 11
3EWK_McMmoS_PASb ---------------------------------------EMILLTDRAGR 11
PA4601_PAS4 MWDEHDRFEGLLGIARDISQQRRAERELRMAATVFEHSTAAIMVTDPAGY 50
PA5442_PAS2 ----------------------------QLAATVFEAASESIVILDPAYN 22
PA0285_PAS2 ----------------------------RQAAAVFDSTREGVLVTDAQAV 22
PA0575_PAS4 ---------------------------LQQAATVFESTAEGVMITDTRQR 23
: * : .: : :. :: : : *
3EWK_McMmoS_PASa ILYANDNFCAVSRYGREELVGQDHRIVNSGYHGKAYIRDMWRTISRGNIW 60
PA1561_PAS1 ITYCNEAFIDISGFSREELMSAPHNLIRHPDVPPAVFAHMWTTLKAGRPW 72
2GJ3_AvNifL_PAS1 ILYANRAFRTITGYGSEEVLGKNESILSNGTTPRLVYQALWGRLAQKKPW 61
3EWK_McMmoS_PASb IIYANPALCRFSGMAEGELLGQSPSILDSPLADQETLAAMQEALQAGQPW 61
PA4601_PAS4 IVQVNDSFSRLTGYSPADVLDQQPRLLTADRQEANQLKHVLASLQHSGSW 100
PA5442_PAS2 LLAVNQAFSRITGYRREDVVGRNASLLVNTPEAQRHYRQIRAELRQHGQW 72
PA0285_PAS2 IVHVNPSFERITGYRSEDVLGKTPAILRSGRQDQAFYQRLWLALREQDVW 72
PA0575_PAS4 ITAVNRAFSEITGYSEQEALGRSPSLLSSGQHDSSFYLAMWNQLERDGHW 73
* . : : * : . . .
3EWK_McMmoS_PASa QGEFCNRRKDG------TRYWVDSTIVPLMDNAGKPRQYISIRRDI--- 100
PA1561_PAS1 MGIVKNRCKNG------DHYWVSAYVTPIYD-QGAVVGYESVRVKPTAE 114
2GJ3_AvNifL_PAS1 SGVLVNRRKDK------TLYLAELTVAPVLNEAGETIYYLGMHRDTSE- 103
3EWK_McMmoS_PASb SGRLLNRRRTGPAPHDAEDYWAEISTTPIHTDGNGLVGYVQIQHD---- 106
PA4601_PAS4 EGEILQKRKTG------ELYPSWVGITAVRDEEGDLVSFVCFFSDISER 143
PA5442_PAS2 QGELVDTRKNG------DPYPQWLQLNLLRDGNGEATHIVGFFTDLSAR 115
PA0285_PAS2 SGEIWNRRKSG------EIYPQWLHIRAVRNDQGQLTHYVGVFSDLSSI 115
PA0575_PAS4 QGEIWNRRKTG------ELYPEWLTISAVHNPQGEITHFVGVFADISTL 116
FAD

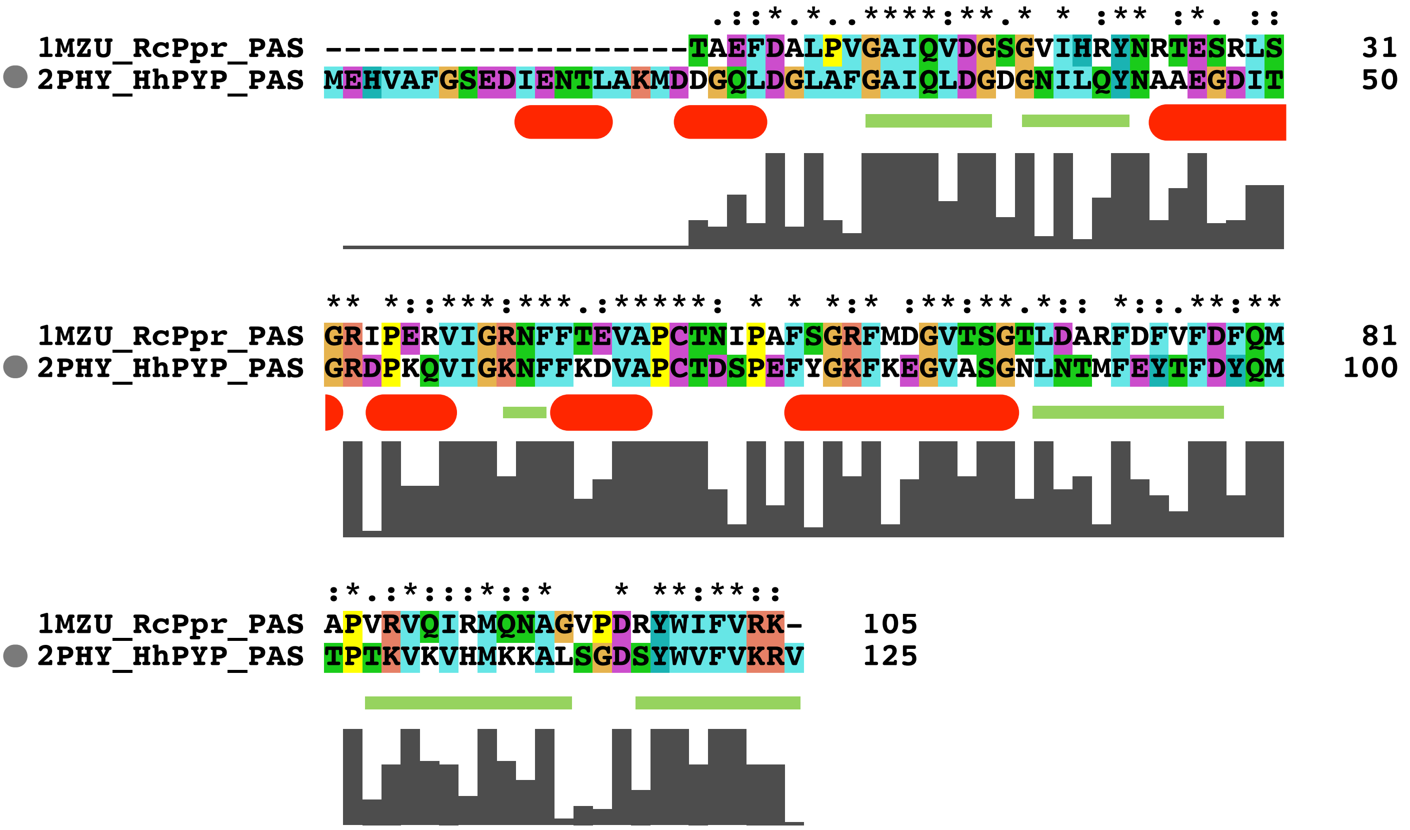
.::*.*..****:**.* * :** :*. ::
1MZU_RcPpr_PAS -------------------TAEFDALPVGAIQVDGSGVIHRYNRTESRLS 31
2PHY_HhPYP_PAS MEHVAFGSEDIENTLAKMDDGQLDGLAFGAIQLDGDGNILQYNAAEGDIT 50
** *::***:***.:*****: * * *:* :**:**.*:: *::.**:**
1MZU_RcPpr_PAS GRIPERVIGRNFFTEVAPCTNIPAFSGRFMDGVTSGTLDARFDFVFDFQM 81
2PHY_HhPYP_PAS GRDPKQVIGKNFFKDVAPCTDSPEFYGKFKEGVASGNLNTMFEYTFDYQM 100
:*.:*:::*::* * **:**::
1MZU_RcPpr_PAS APVRVQIRMQNAGVPDRYWIFVRK- 105
2PHY_HhPYP_PAS TPTKVKVHMKKALSGDSYWVFVKRV 125

HA

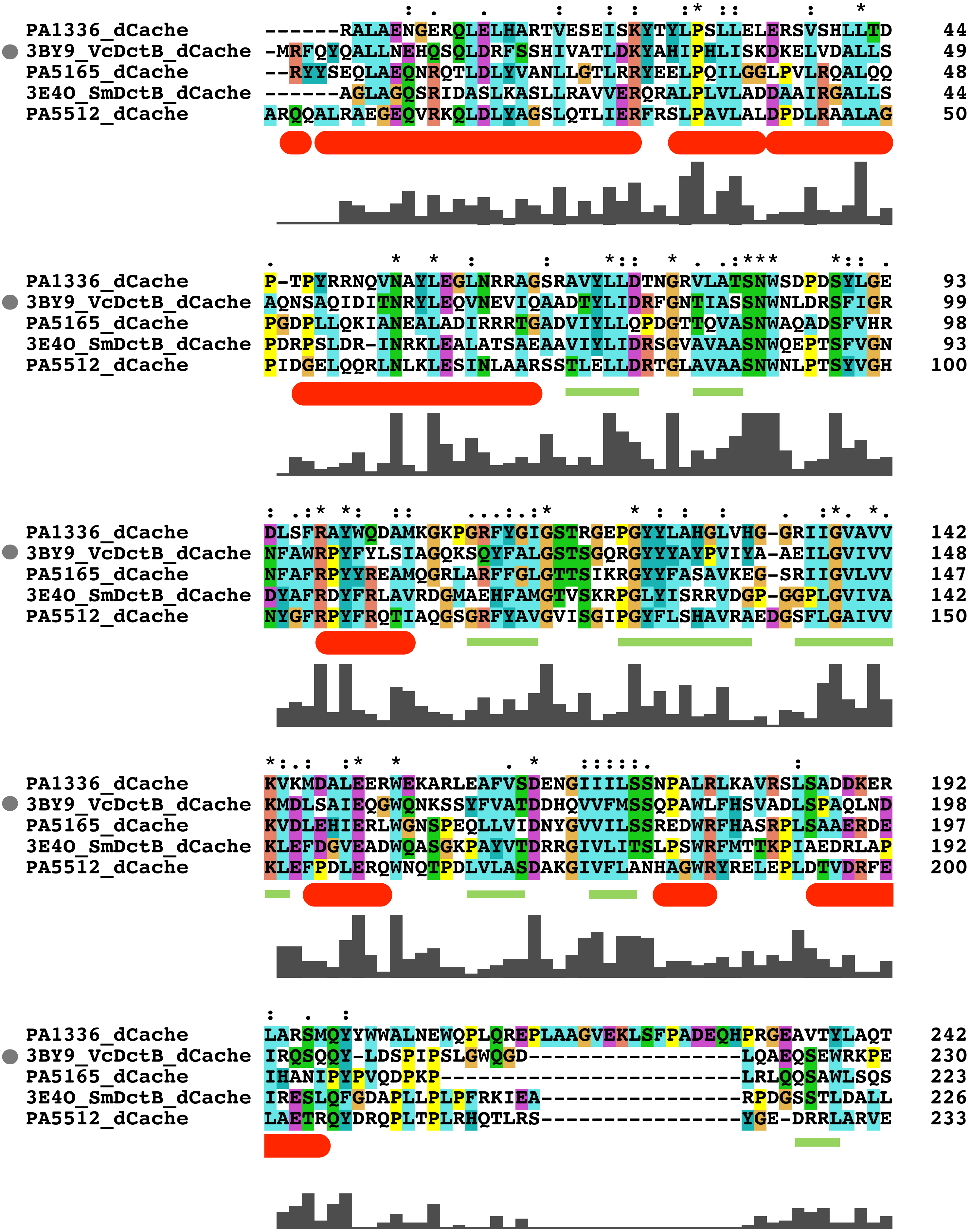

dCache carboxylic acids

```
                                  :
  PA1336_dCache        RALNDTPWNLTLLSPLEDLRRDAVRNGMLAAIGFALLAFLLIAWNERRKV    292
● 3BY9_VcDctB_dCache   KHWLQDDYIVSSRPLPELALTIRVLSPKI---------------------    259
  PA5165_dCache        RTLPETGWTVS-IYAPRTLIERPVRS------------------------    248
  3E4O_SmDctB_dCache   PGDSTAAFLRVETMVPSTNWRLEQLSPL----------------------    254
  PA5512_dCache        SADGEKDYLWQSLDLPNDGWTLHLLRDTASIQDDV---------------    268
```

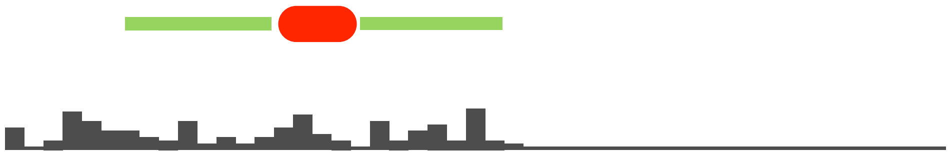

```
  PA1336_dCache        LATR    296
● 3BY9_VcDctB_dCache   ----    259
  PA5165_dCache        ----    248
  3E4O_SmDctB_dCache   ----    254
  PA5512_dCache        ----    268
```

dCache carboxylic acids

```
1P0Z_KpCitA_sCache    --------------------------------------------------
3BY8_EcDcuS_sCache    --------------------------------------------------
PA4021_PAS1           --------------------------------------------------
PA4147_PAS1           --------------------------------------------------
4EXO_VP0183_sCache    --------------------------------------------------
PA2652_sCache         ---------KDTRERLLGDRRAELEHYVQIAMGSIQAEYDRSANGDLN--    39
4K08_Adeh3718_sCache  --------------------------------------------------
5G4Y_PsCdsD_sCache    SGLVPRGSHMQTHEDLYRAKSEKTMHVVQTASGILTFYQGLEAAGSMTRE    50

                                          .   *                ;
1P0Z_KpCitA_sCache    -----------------ATYITVGDASGQRLYHV-NPDEIGKSMEGGDSD    32
3BY8_EcDcuS_sCache    -----------------LLFIVVTDMQSLRYSHP-EAQRIGQPFKGDDIL    32
PA4021_PAS1           -------HSLYQQLSGNDHAVLLTDARG-VILNCVNADAQRQTFEHAGLW    42
PA4147_PAS1           ---------LHGRVRDADYCVLLTDAQG-RTIDYRVESTIRNDCRKAGLY    40
4EXO_VP0183_sCache    ----------------SDGYFFAYDSQGINTLHAIKPSLEGKNLYDLKDE    34
PA2652_sCache         -ARAEAIARLSKIKYGKDGYIFGYDSQVVRLFRGDSPVDVGKSFRDRRDP    88
4K08_Adeh3718_sCache  -------------------YFWVNDLEPRMVMHPTNPQLDGQDLSGYRDP    31
5G4Y_PsCdsD_sCache    AAQQQALKEIKGLRYSQNDYFWINDLRPVMIMHPTNPKLEGQDISTIKDP   100

                       .                                    .
1P0Z_KpCitA_sCache    EALINAKSYVSVRK--GSLGSSLRGKSP-IQDATGKVIG----------I    69
3BY8_EcDcuS_sCache    KALN-GEENVAINR--GFLAQALRVFTP-IYDENHKQIG----------V    68
PA4021_PAS1           LGADWSEPCEGTNGIGTCVVERQALTIQQEEHFRSRHTGLTCSASPVFDP    92
PA4147_PAS1           LGTCWSEGEEGTCGVAAVLTDRTAVTVHKRDHFRAAFIDLTCSAAPVFDP    90
4EXO_VP0183_sCache    NGVAVIAGLIDASQ-KGDGFLYFSWHKP-TINAQAPKLGYAEYLQKWDWV    82
PA2652_sCache         SGVYLNRELVEAGR-NGSHYVTYTSPLPGNESVMVPKLSYTLYLPKWDMV   137
4K08_Adeh3718_sCache  NGKLLFQEFVRTVRARGSGFVDYLWPKP-GSTVPVPKISFVTQYQPWGWV    80
5G4Y_PsCdsD_sCache    DGFAVFNEMVALVKSKGAGMVNYRWPKP-GASEPVKKTSYVQLFQPWGWI   149

                       .    :
1P0Z_KpCitA_sCache    VSVGYTIE------------    77
3BY8_EcDcuS_sCache    VAIGLEL-------------    75
PA4021_PAS1           QGELMAVLDVSSAR------   106
PA4147_PAS1           QGELLGVLDVSAVQSPDDRR   110
4EXO_VP0183_sCache    LGTGIYIDD-----------    91
PA2652_sCache         IGSAINLDGVEAQLVEIKQD   157
4K08_Adeh3718_sCache  VGSGLYVD------------    88
5G4Y_PsCdsD_sCache    LGSGVYVDDVAAEFKTQLW-   168
```

sCache carboxylic acids

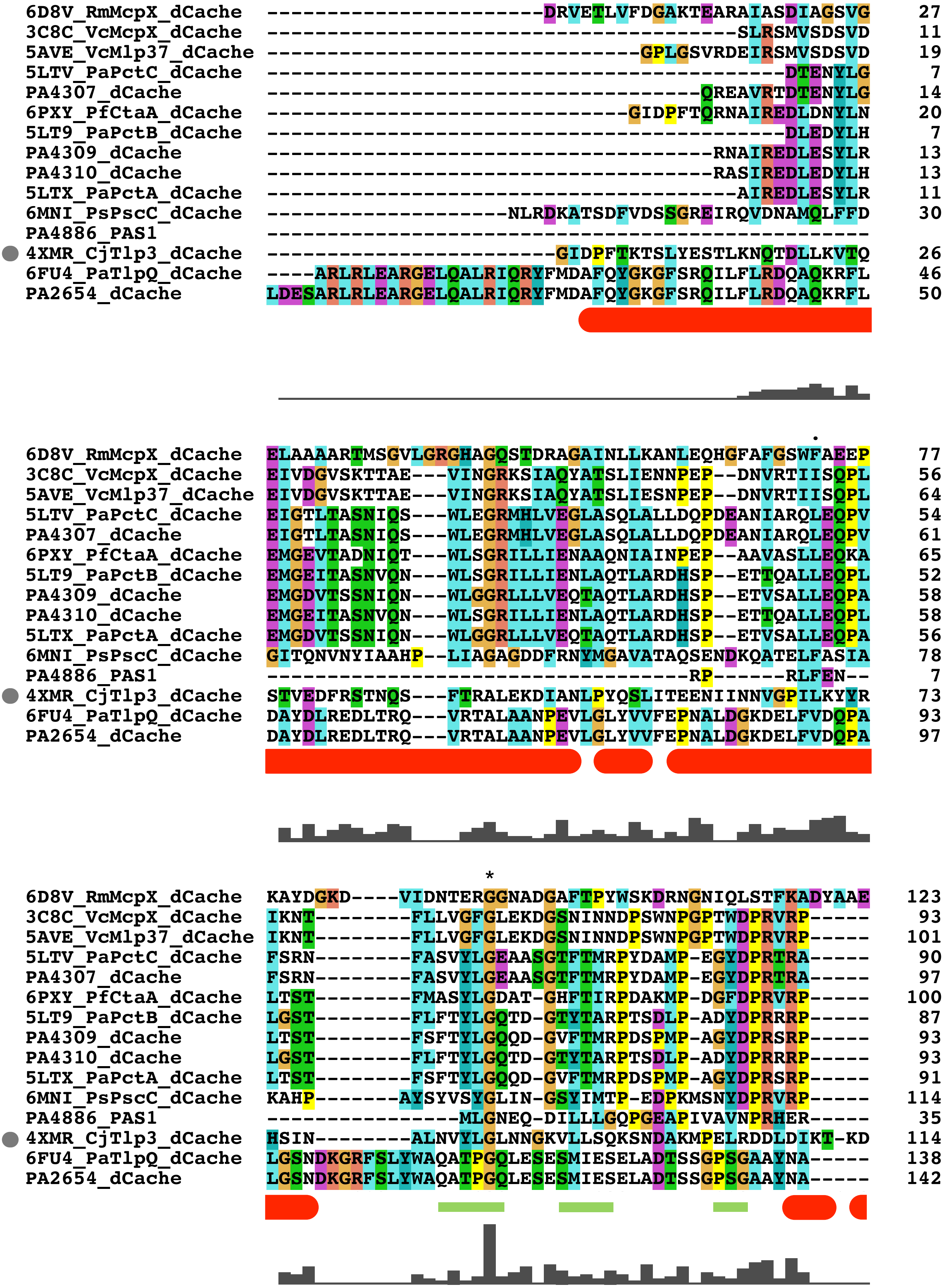
6D8V_RmMcpX_dCache -----------------------DRVETLVFDGAKTEARAIASDIAGSVG 27
3C8C_VcMcpX_dCache ---------------------------------------SLRSMVSDSVD 11
5AVE_VcMlp37_dCache -------------------------------GPLGSVRDEIRSMVSDSVD 19
5LTV_PaPctC_dCache ---DTENYLG 7
PA4307_dCache ------------------------------------QREAVRTDTENYLG 14
6PXY_PfCtaA_dCache ------------------------------GIDPFTQRNAIREDLDNYLN 20
5LT9_PaPctB_dCache ---DLEDYLH 7
PA4309_dCache -------------------------------------RNAIREDLESYLR 13
PA4310_dCache -------------------------------------RASIREDLEDYLH 13
5LTX_PaPctA_dCache ---------------------------------------AIREDLESYLR 11
6MNI_PsPscC_dCache --------------------NLRDKATSDFVDSSGREIRQVDNAMQLFFD 30
PA4886_PAS1 --
4XMR_CjTlp3_dCache ------------------------GIDPFTKTSLYESTLKNQTDLLKVTQ 26
6FU4_PaTlpQ_dCache ----ARLRLEARGELQALRIQRYFMDAFQYGKGFSRQILFLRDQAQKRFL 46
PA2654_dCache LDESARLRLEARGELQALRIQRYFMDAFQYGKGFSRQILFLRDQAQKRFL 50
6D8V_RmMcpX_dCache ELAAAARTMSGVLGRGHAGQSTDRAGAINLLKANLEQHGFAFGSWFAEEP 77
3C8C_VcMcpX_dCache EIVDGVSKTTAE---VINGRKSIAQYATSLIENNPEP--DNVRTIISQPL 56
5AVE_VcMlp37_dCache EIVDGVSKTTAE---VINGRKSIAQYATSLIESNPEP--DNVRTIISQPL 64
5LTV_PaPctC_dCache EIGTLTASNIQS---WLEGRMHLVEGLASQLALLDQPDEANIARQLEQPV 54
PA4307_dCache EIGTLTASNIQS---WLEGRMHLVEGLASQLALLDQPDEANIARQLEQPV 61
6PXY_PfCtaA_dCache EMGEVTADNIQT---WLSGRILLIENAAQNIAINPEP--AAVASLLEQKA 65
5LT9_PaPctB_dCache EMGEITASNVQN---WLSGRILLIENLAQTLARDHSP--ETTQALLEQPL 52
PA4309_dCache EMGDVTSSNIQN---WLGGRLLLVEQTAQTLARDHSP--ETVSALLEQPA 58
PA4310_dCache EMGEITASNVQN---WLSGRILLIENLAQTLARDHSP--ETTQALLEQPL 58
5LTX_PaPctA_dCache EMGDVTSSNIQN---WLGGRLLLVEQTAQTLARDHSP--ETVSALLEQPA 56
6MNI_PsPscC_dCache GITQNVNYIAAHP--LIAGAGDDFRNYMGAVATAQSENDKQATELFASIA 78
PA4886_PAS1 -------------------------------------RP------RLFEN-- 7
4XMR_CjTlp3_dCache STVEDFRSTNQS---FTRALEKDIANLPYQSLITEENIINNVGPILKYYR 73
6FU4_PaTlpQ_dCache DAYDLREDLTRQ---VRTALAANPEVLGLYVVFEPNALDGKDELFVDQPA 93
PA2654_dCache DAYDLREDLTRQ---VRTALAANPEVLGLYVVFEPNALDGKDELFVDQPA 97
6D8V_RmMcpX_dCache KAYDGKD----VIDNTERGGNADGAFTPYWSKDRNGNIQLSTFKADYAAE 123
3C8C_VcMcpX_dCache IKNT--------FLLVGFGLEKDGSNINNDPSWNPGPTWDPRVRP----- 93
5AVE_VcMlp37_dCache IKNT--------FLLVGFGLEKDGSNINNDPSWNPGPTWDPRVRP----- 101
5LTV_PaPctC_dCache FSRN--------FASVYLGEAASGTFTMRPYDAMP-EGYDPRTRA----- 90
PA4307_dCache FSRN--------FASVYLGEAASGTFTMRPYDAMP-EGYDPRTRA----- 97
6PXY_PfCtaA_dCache LTST--------FMASYLGDAT-GHFTIRPDAKMP-DGFDPRVRP----- 100
5LT9_PaPctB_dCache LGST--------FLFTYLGQTD-GTYTARPTSDLP-ADYDPRRRP----- 87
PA4309_dCache LTST--------FSFTYLGQQD-GVFTMRPDSPMP-AGYDPRSRP----- 93
PA4310_dCache LGST--------FLFTYLGQTD-GTYTARPTSDLP-ADYDPRRRP----- 93
5LTX_PaPctA_dCache LTST--------FSFTYLGQQD-GVFTMRPDSPMP-AGYDPRSRP----- 91
6MNI_PsPscC_dCache KAHP-------AYSYVSYGLIN-GSYIMTP-EDPKMSNYDPRVRP----- 114
PA4886_PAS1 ----------------MLGNEQ-DILLLGQPGEAPIVAVNPRHER----- 35
4XMR_CjTlp3_dCache HSIN--------ALNVYLGLNNGKVLLSQKSNDAKMPELRDDLDIKT-KD 114
6FU4_PaTlpQ_dCache LGSNDKGRFSLYWAQATPGQLESESMIESELADTSSGPSGAAYNA----- 138
PA2654_dCache LGSNDKGRFSLYWAQATPGQLESESMIESELADTSSGPSGAAYNA----- 142

Amino acids

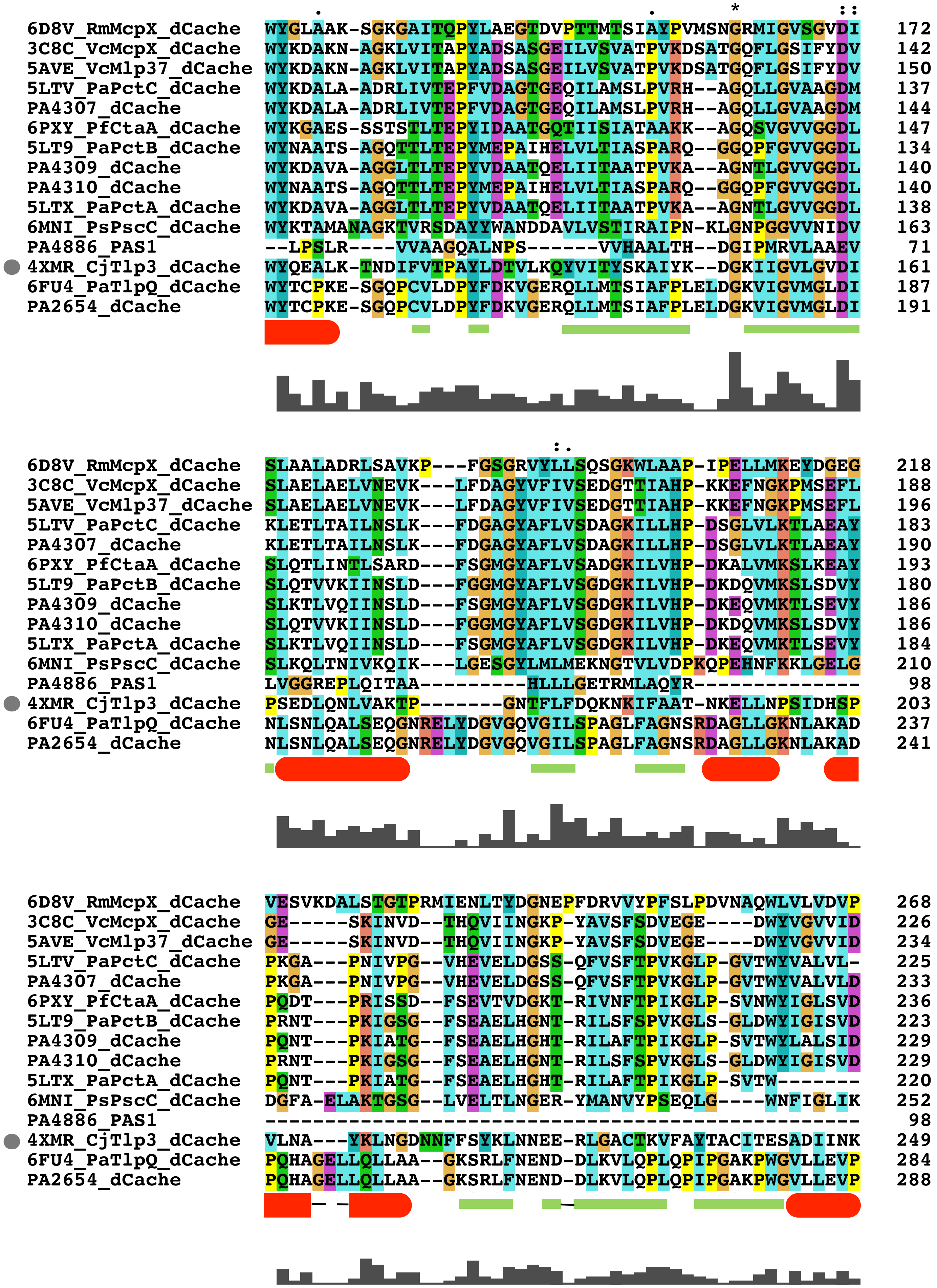

6D8V_RmMcpX_dCache WYGLAAK-SGKGAITQPYLAEGTDVPTTMTSIAYPVMSNGRMIGVSGVDI 172
3C8C_VcMcpX_dCache WYKDAKN-AGKLVITAPYADSASGEILVSVATPVKDSATGQFLGSIFYDV 142
5AVE_VcMlp37_dCache WYKDAKN-AGKLVITAPYADSASGEILVSVATPVKDSATGQFLGSIFYDV 150
5LTV_PaPctC_dCache WYKDALA-ADRLIVTEPFVDAGTGEQILAMSLPVRH--AGQLLGVAAGDM 137
PA4307_dCache WYKDALA-ADRLIVTEPFVDAGTGEQILAMSLPVRH--AGQLLGVAAGDM 144
6PXY_PfCtaA_dCache WYKGAES-SSTSTLTEPYIDAATGQTIISIATAAKK--AGQSVGVVGGDL 147
5LT9_PaPctB_dCache WYNAATS-AGQTTLTEPYMEPAIHELVLTIASPARQ--GGQPFGVVGGDL 134
PA4309_dCache WYKDAVA-AGGLTLTEPYVDAATQELIITAATPVKA--AGNTLGVVGGDL 140
PA4310_dCache WYNAATS-AGQTTLTEPYMEPAIHELVLTIASPARQ--GGQPFGVVGGDL 140
5LTX_PaPctA_dCache WYKDAVA-AGGLTLTEPYVDAATQELIITAATPVKA--AGNTLGVVGGDL 138
6MNI_PsPscC_dCache WYKTAMANAGKTVRSDAYYWANDDAVLVSTIRAIPN-KLGNPGGVVNIDV 163
PA4886_PAS1 --LPSLR----VVAAGQALNPS------VVHAALTH--DGIPMRVLAAEV 71
4XMR_CjTlp3_dCache WYQEALK-TNDIFVTPAYLDTVLKQYVITYSKAIYK--DGKIIGVLGVDI 161
6FU4_PaTlpQ_dCache WYTCPKE-SGQPCVLDPYFDKVGERQLLMTSIAFPLELDGKVIGVMGLDI 187
PA2654_dCache WYTCPKE-SGQPCVLDPYFDKVGERQLLMTSIAFPLELDGKVIGVMGLDI 191

6D8V_RmMcpX_dCache SLAALADRLSAVKP---FGSGRVYLLSQSGKWLAAP-IPELLMKEYDGEG 218
3C8C_VcMcpX_dCache SLAELAELVNEVK---LFDAGYVFIVSEDGTTIAHP-KKEFNGKPMSEFL 188
5AVE_VcMlp37_dCache SLAELAELVNEVK---LFDAGYVFIVSEDGTTIAHP-KKEFNGKPMSEFL 196
5LTV_PaPctC_dCache KLETLTAILNSLK---FDGAGYAFLVSDAGKILLHP-DSGLVLKTLAEAY 183
PA4307_dCache KLETLTAILNSLK---FDGAGYAFLVSDAGKILLHP-DSGLVLKTLAEAY 190
6PXY_PfCtaA_dCache SLQTLINTLSARD---FSGMGYAFLVSADGKILVHP-DKALVMKSLKEAY 193
5LT9_PaPctB_dCache SLQTVVKIINSLD---FGGMGYAFLVSGDGKILVHP-DKDQVMKSLSDVY 180
PA4309_dCache SLKTLVQIINSLD---FSGMGYAFLVSGDGKILVHP-DKEQVMKTLSEVY 186
PA4310_dCache SLQTVVKIINSLD---FGGMGYAFLVSGDGKILVHP-DKDQVMKSLSDVY 186
5LTX_PaPctA_dCache SLKTLVQIINSLD---FSGMGYAFLVSGDGKILVHP-DKEQVMKTLSEVY 184
6MNI_PsPscC_dCache SLKQLTNIVKQIK---LGESGYLMLMEKNGTVLVDPKQPEHNFKKLGELG 210
PA4886_PAS1 LVGGREPLQITAA---------HLLLGETRMLAQYR-------------- 98
4XMR_CjTlp3_dCache PSEDLQNLVAKTP-------GNTFLFDQKNKIFAAT-NKELLNPSIDHSP 203
6FU4_PaTlpQ_dCache NLSNLQALSEQGNRELYDGVGQVGILSPAGLFAGNSRDAGLLGKNLAKAD 237
PA2654_dCache NLSNLQALSEQGNRELYDGVGQVGILSPAGLFAGNSRDAGLLGKNLAKAD 241

6D8V_RmMcpX_dCache VESVKDALSTGTPRMIENLTYDGNEPFDRVVYPFSLPDVNAQWLVLVDVP 268
3C8C_VcMcpX_dCache GE-----SKINVD--THQVIINGKP-YAVSFSDVEGE----DWYVGVVID 226
5AVE_VcMlp37_dCache GE-----SKINVD--THQVIINGKP-YAVSFSDVEGE----DWYVGVVID 234
5LTV_PaPctC_dCache PKGA---PNIVPG--VHEVELDGSS-QFVSFTPVKGLP-GVTWYVALVL- 225
PA4307_dCache PKGA---PNIVPG--VHEVELDGSS-QFVSFTPVKGLP-GVTWYVALVLD 233
6PXY_PfCtaA_dCache PQDT---PRISSD--FSEVTVDGKT-RIVNFTPIKGLP-SVNWYIGLSVD 236
5LT9_PaPctB_dCache PRNT---PKIGSG--FSEAELHGNT-RILSFSPVKGLS-GLDWYIGISVD 223
PA4309_dCache PQNT---PKIATG--FSEAELHGHT-RILAFTPIKGLP-SVTWYLALSID 229
PA4310_dCache PRNT---PKIGSG--FSEAELHGNT-RILSFSPVKGLS-GLDWYIGISVD 229
5LTX_PaPctA_dCache PQNT---PKIATG--FSEAELHGHT-RILAFTPIKGLP-SVTW------- 220
6MNI_PsPscC_dCache DGFA-ELAKTGSG--LVELTLNGER-YMANVYPSEQLG----WNFIGLIK 252
PA4886_PAS1 -- 98
4XMR_CjTlp3_dCache VLNA---YKLNGDNNFFSYKLNNEE-RLGACTKVFAYTACITESADIINK 249
6FU4_PaTlpQ_dCache PQHAGELLQLLAA--GKSRLFNEND-DLKVLQPLQPIPGAKPWGVLLEVP 284
PA2654_dCache PQHAGELLQLLAA--GKSRLFNEND-DLKVLQPLQPIPGAKPWGVLLEVP 288

Amino acids

6D8V_RmMcpX_dCache	R--------------	269
3C8C_VcMcpX_dCache	---------------	226
5AVE_VcMlp37_dCache	---------------	234
5LTV_PaPctC_dCache	---------------	225
PA4307_dCache	RDTAYSMLS------	242
6PXY_PfCtaA_dCache	KDKAFSMLSEFRTS	250
5LT9_PaPctB_dCache	K--------------	224
PA4309_dCache	KDKAYAMLSK-----	239
PA4310_dCache	KDKAYAMLTKL---	240
5LTX_PaPctA_dCache	---------------	220
6MNI_PsPscC_dCache	Q--------------	253
PA4886_PAS1	---------------	98
4XMR_CjTlp3_dCache	PIYKA----------	254
6FU4_PaTlpQ_dCache	K--------------	285
PA2654_dCache	KSALLGPAL------	297

Amino acids

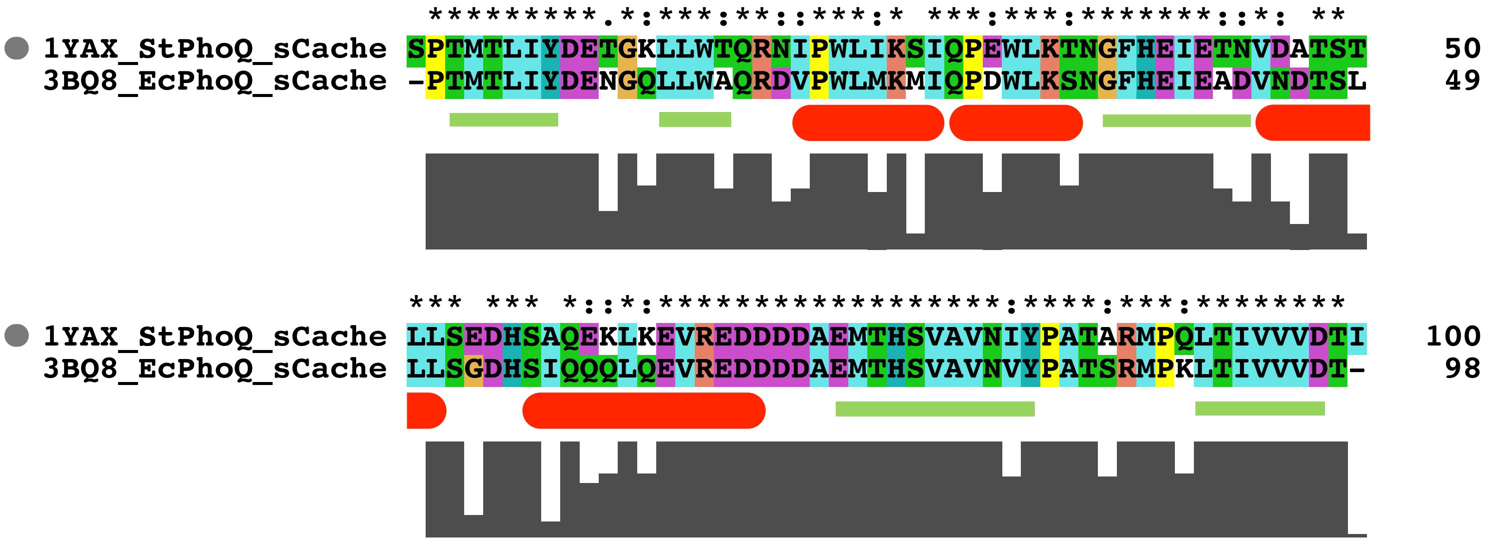

Metals (sCache)

```
4M4X_MmAhR_PASa       ------NGFVLVVTAD-ALVFYASSTIQDYLGFQQSDVIHQSVYELIHT-   42
5NJ8_HsAhR_PASa       --------FVLVVTTD-ALVFYASSTIQDYLGFQQSDVIHQSVYELIHT-   40
5Y7Y_HsAhRR_PASa      ------NGFALVVSAE-GTIFYASATIVDYLGFHQTDVMHQNIYDYIHV-   42
5Y7Y_BtARNT_PASa      ------DGFLFIVSCETGRVVYVSDSVTPVLNQPQSEWFGSTLYDQVHP-   43
5Y7Y_BtARNT_PASb      ---------------TEFISRHNIEGIFTFVDHRCVATVG--YQPQE---   30
4ZP4_MmARNT_PASa      -----ADGFLFIVSCETGRVVYVSDSVTPVLNQPQSEWFGSTLYDQVHP-   44
4ZP4_MmARNT_PASb      ----------------EFISRHNIEGIFTFVDHRCVATVG--YQPQE---   29
1X0O_HsARNT_PASb      ------GAMDNVCQPTEFISRHNIEGIFTFVDHRCVATVG--YQPQE---   39
4F3L_MmBMAL1_PASB     --------------SMEYVSRHAIDGKFVFVDQRATAILA--YLPQE---   31
4F3L_MmClock_PASb     ------------EPNEEFTSRHSLEWKFLFLDHRAPPIIG--YLPFE---   33
PA1181_PAS2           -----ERIKLATDAGQIGIWEWDLARNRLHWDFRMFDLYG--IRSGPGE-   42
PA4112_PAS3           -----DQLQMAADVARLGIWRWNLADDSLQWNERMCEMYGQPLALRDGG-   44
PA5442_PAS1           ---SEARLAMALEASELGLWDWDLESGRVQHS--YLEAVF--SDAGDGP-   42
PA0285_PAS1           ---SEQRLNRALEAVRDGLWDWDLVTDRMFVSPGYAALIG--LAPEELG-   44
PA0338_PAS1           ---LEKTLRYALEIVSDGIWDWNIATNQVSRSAGWYLMLG--YPPHSLP-   44
1ZTU_DrBphp_PAS1      ------HGALLTADGHSGEVLQMSLNAATFLGQEPTVLRGQTLAALLP--   42
5HSQ_AfAgp1_PAS       -GAIQEHGALLVLSAREFSVVQASDNLANYIG--VDLPIG-AVATEANL-   45
5AKP_XccBphP_nPAS     ------YGVLLVIDPADGRIVQASTTAADLLGVPMAALLGMPYTQVLTL-   43
3C2W_PaBphP_PAS1      -----PHGALVTLRADGMVLAAS-ENIQALLG-----FVASPGSYLTQE-   38
PA4117_PAS1           ---IQPHGALVTLRADGMVLAAS-ENIQALLG-----FVASPGSYLTQE-   40
6G1Y_AfAgp2_PAS       PGYIQPHGCLIACDNAMRMVLRHSENCGELLG-LEGDLNGRTAEDVLGK-   48
4GW9_RpBphP1_PAS1     ------HGALLVVSEPDHRIIQASANAAEFLN--LGSVLGVPLAE-IDG-   40
4GW9_RpBphP1_PAS2     ------QPVLITDAEGRILLMNDSFRDMLPAGSPSAVHLDDLAGFFVESN   44
2OOL_RpBphP3_PAS1     ------HGYLFVVSETDLRIASVSANVEDLLRQPPASLLNVPIAHYLTA-   43
4E04_RpBphP2_PAS      ------HG-LLLALAADMTIVAGSDNLPELTGLAIGALIGRSAADVFDS-   42
6PTQ_SaBphP2_PAS      ------HGVLLAFRGPDRLLEVVSANAQALLGRPPETLLGQPVGRVLPA-   43
2VEA_SsCph1_PAS1      ------HGLVVVLQEPDLTISQISANCTGILGRSPEDLLGRTLGEVFDS-   43
6BAF_SaBphP_PAS       ------HGVLLVLSEPGLVLTHASENAPAVLGNSAEQLLGAPLGHFIEP-   43
6JT0_HsSGCa_PAS       ------FHFMFDKDMTILQFGNGIRRLMNRRDFQGKPNFEEYFEILTPK-   43
6JT0_HsSGCb_PAS       ----FPFHIIFDRDLVVTQCGNAIYRVLPQLQ-PGNCSLLSVFSLVRPH-   44
4GJ4_MsSGC_PAS        ----FPWHFITDKRLELVQLGAGFMRLFGTHLATHGSSLGTYFRLLRPR-   45
1OJ5_MmNCoA-1_PAS1    --------------VESFMTKQDTTGKIISIDTSSLRAAG--RTGWED--   32
5NWM_HsNocoA1_PASb    ---------GHMTGVESFMTKQDTTGKIISIDTSSLRAAG--RTGWED--   37
4HH2_RsPpsR_nPAS      -------DISLLVSQEGVVREVMANPHHPSFG-QLSEWEGRPLEEVLTA-   41
4HH2_RsPpsR_PAS1      --------PMVLVSMSTGRIVDLNSAAGLLLGGVRQDLLGAAIAQEFEG-   41
4HH2_RsPpsR_PAS2      -------DGIVFSDADGTIRGANEAFLNMTDSSSLAAIRGRSIADFLAR-   42
4LRX_EcDhR_PAS        ------------------------DGVISWDEQGNLQFINAQAARVLRL-   25
PA5124_PAS1           -----------TDTLHRLLLDNLTTAVILLNGELRLEYMNPAAEMLLAV-   38
5AKP_XccBphP_cPAS     ---DGVAIIERGTANAAHRLLFVNTAFADVCGSDVAELIGRELQTLYAS-   46
5LLW_ISPadC_PAS       ------FGAMLIVEKDTQQIVYASANSAEYFSVADNTIHE--LSDIKQA-   41
```

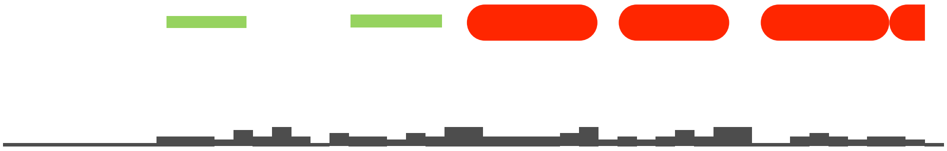

no cofactor or ligand binding

```
4M4X_MmAhR_PASa     EDRAEFQRQLHWALNP---------DSAQGVDEAHGPPQAAVYYTPDQLP   83
5NJ8_HsAhR_PASa     EDRAEFQRQLHWALNPS-----QCTESGQGIEEATGLPQTVVCYNPDQIP   85
5Y7Y_HsAhRR_PASa    DDRQDFCRQLHWAMDPPQVVFGQPPPLETGDDAILGRLLRAQEWGTG-TP   91
5Y7Y_BtARNT_PASa    DDVDKLREQLSTSEN-----------------------------------   58
5Y7Y_BtARNT_PASb    LLGKNIVEFCHPEDQQ-----------------------------LLRDS   51
4ZP4_MmARNT_PASa    DDVDKLREQLSTSENAL-------------TGRVLDLKTGTVKKEGQQSS   81
4ZP4_MmARNT_PASb    LLGKNIVEFCHPEDQQ-----------------------------LLRDS   50
1X0O_HsARNT_PASb    LLGKNIVEFCHPEDQQ-----------------------------LLRDS   60
4F3L_MmBMAL1_PASB   LLGTSCYEYFHQDDIG-----------------------------HLAEC   52
4F3L_MmClock_PASb   VLGTSGYDYYHVDDLE-----------------------------NLAKC   54
PA1181_PAS2         TTVEQWKASLHAEDHD-----------------------------RVLRE   63
PA4112_PAS3         LVYEHWRSRLHPEDLE-----------------------------RTEAS   65
PA5442_PAS1         EDYRRLIESIHPGDLP-----------------------------RIRRA   63
PA0285_PAS1         DPIETWKKRLHPEEYA-----------------------------TVLEA   65
PA0338_PAS1         ESVETWKSIIHPEDYP-----------------------------RVMAS   65
1ZTU_DrBphp_PAS1    EQWPALQAALPPGCP---------------------------------DA   59
5HSQ_AfAgp1_PAS     PFISVLSAWYSGAAS----------------------------------N   61
5AKP_XccBphP_nPAS   PEAQPFAVDDQPQHL--------------------------------MHA   61
3C2W_PaBphP_PAS1    QVGPEVLRMLEEG-L------------------------------TGNGP   57
PA4117_PAS1         QVGPEVLRMLEEG-L------------------------------TGNGP   59
6G1Y_AfAgp2_PAS     KLVHDLRNALTVTGRT--------------------------------TR   66
4GW9_RpBphP1_PAS1   DLLIKILPHLDPT--------------------------------AEGMP   58
4GW9_RpBphP1_PAS2   DFLRNVAELIDHGRG-------------------------------WRGE   63
2OOL_RpBphP3_PAS1   ASAARLTHALHGGDP------------------------------AAINP   63
4E04_RpBphP2_PAS    ETHNRLTIALAEPGA------------------------------AVGAP   62
6PTQ_SaBphP2_PAS    EVLAQWEPLVARGSV------------------------------RVVLP   63
2VEA_SsCph1_PAS1    FQIDPIQSRLTAGQI------------------------------SSLNP   63
6BAF_SaBphP_PAS     SVREPLEADLRSARL------------------------------KQLNP   63
6JT0_HsSGCa_PAS     -INQTFSGIMTMLNMQ--------------------------------FV   60
6JT0_HsSGCb_PAS     -IDISFHGILSHINTV--------------------------------FV   61
4GJ4_MsSGC_PAS      GVPLDFREILKRVNTP--------------------------------FM   63
1OJ5_MmNCoA-1_PAS1  LVRKCIYAFFQPQGREP----------------------------SYARQ   54
5NWM_HsNocoA1_PASb  LVRKCIYAFFQPQGREP----------------------------SYARQ   59
4HH2_RsPpsR_nPAS    ESVAKFRLRSEGLEPG-----------------------------RGSVA   62
4HH2_RsPpsR_PAS1    RRRGEFMETMTNLAAT-----------------------------ESAAP   62
4HH2_RsPpsR_PAS2    -GSVDLRVLIDSVRRTG---------------------------QLRLYA   64
4LRX_EcDhR_PAS      DATASQGRAITELLT-----------------------------LP---A   43
PA5124_PAS1         SGQRSHGQFISELFTE----------------------------SPEALN   60
5AKP_XccBphP_cPAS   DAPRANVELLQDALRNG---------------------------RAAYVT   69
5LLW_ISPadC_PAS     NINSLLPEHLISGLAS---------------------------------A   58
```

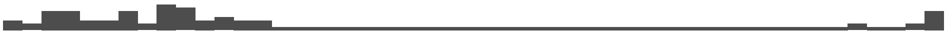

no cofactor or ligand binding

```
4M4X_MmAhR_PASa       PENASFMERCFRCRLRCLLDN--------------------------SSG   107
5NJ8_HsAhR_PASa       PENSPLMERCFICRLRCLLDN--------------------------SSG   109
5Y7Y_HsAhRR_PASa      TEYSAFLTRCFICRVRCLLDS--------------------------TSG   115
5Y7Y_BtARNT_PASa      ----MGSRRSFICRMRCGN-----------------------------PH    75
5Y7Y_BtARNT_PASb      FQQVVKLKGQVLSVMFRFRSK--------------------------NRE    75
4ZP4_MmARNT_PASa      MRMCMGSRRSFICRMRCGTSSVDPVSMNRLSFLRNRCRNGLGSVKEGEPH   131
4ZP4_MmARNT_PASb      FQQVVKLKGQVLSVMFRFRSK--------------------------TRE    74
1X0O_HsARNT_PASb      FQQVVKLKGQVLSVMFRFRSK--------------------------NQE    84
4F3L_MmBMAL1_PASB     HRQVLQTREKITTNCYKFKIK--------------------------DGS    76
4F3L_MmClock_PASb     HEHLMQY-GKGKSCYYRFLTK--------------------------GQQ    77
PA1181_PAS2           LERAVSGLQ-KFDCEFRIVRP--------------------------NRE    86
PA4112_PAS3           LRAAVEGRG-NYDVIFRVVLP--------------------------DGG    88
PA5442_PAS1           LAEHLKGRSELYRVEYRVRDR--------------------------EGG    87
PA0285_PAS1           HRNHLQGLTDNLDHIYRLRHK--------------------------DGD    89
PA0338_PAS1           FQAYLDGESPEYCEEYRCRTY--------------------------SGD    89
1ZTU_DrBphp_PAS1      LQYRATLDWPAAGHLSLTVHR--------------------------VGE    83
5HSQ_AfAgp1_PAS       FRYAWAEK-----KLDVSAHR--------------------------SGT    80
5AKP_XccBphP_nPAS     EVRFPQRATPPASAWVAAWHL--------------------------YPQ    85
3C2W_PaBphP_PAS1      WSNSVETRIGE-HLFDVIGHS--------------------------YKE    80
PA4117_PAS1           WSNSVETRIGE-HLFDVIGHS--------------------------YKE    82
6G1Y_AfAgp2_PAS       PAMLPAMETSDGRSFDISLHR--------------------------YKS    90
4GW9_RpBphP1_PAS1     VAVRCRIGNPS-TEYDGLMHRP-------------------------PEG    82
4GW9_RpBphP1_PAS2     VLLRGAGNRPLPLAVRADPVT--------------------------RTE    87
2OOL_RpBphP3_PAS1     IRLDVVTPDGE-RAFNGILHR--------------------------HDS    86
4E04_RpBphP2_PAS      IAVGFTMPDGE-RAFNGSWHR--------------------------HDQ    85
6PTQ_SaBphP2_PAS      AG-----------AYRALLHE--------------------------SDG    76
2VEA_SsCph1_PAS1      SKLWARVMGDDFVIFDGVFHRN-------------------------SDG    88
6BAF_SaBphP_PAS       LKVVWRVDGVD-RFFDGIAHR--------------------------HQG    86
6JT0_HsSGCa_PAS       VRVRR---------WDNSVK----------------------------KS    73
6JT0_HsSGCb_PAS       LRSKEGLLDVEKLECEDELTG---------------------------TE    84
4GJ4_MsSGC_PAS        FALKMPG-----------ST----------------------------AL    74
1OJ5_MmNCoA-1_PAS1    LFQEVMTRGTASSPSYRFILN--------------------------DGT    78
5NWM_HsNocoA1_PASb    LFQEVMTRGTASSPSYRFILN--------------------------DGT    83
4HH2_RsPpsR_nPAS      VELNHIDPRSFEFPIRYILHR-------------------------LPAD    87
4HH2_RsPpsR_PAS1      VEVLARRSQKRLLVVPRVFRAA-------------------------GER    87
4HH2_RsPpsR_PAS2      TRLTTDFAGQIAAEISATWLD--------------------------DRE    88
4LRX_EcDhR_PAS        VLQQAIKQAHPLKHVEATFES---------------------------QH    66
PA5124_PAS1           SLRQAVEQAHPFTKREATLTS---------------------------IT    83
5AKP_XccBphP_cPAS     LPLQVSDGAPVYRQFHLEPLP--------------------------SPS    93
5LLW_ISPadC_PAS       IRENEPIWVET-DRLSFLGWR--------------------------HEN    81
```

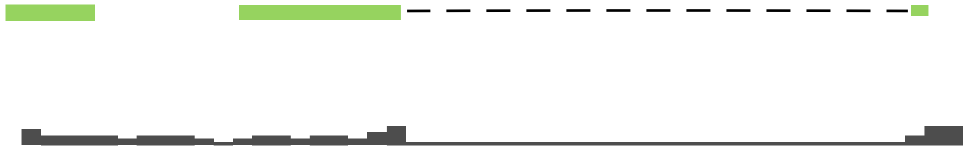

no cofactor or ligand binding

```
4M4X_MmAhR_PASa     FLAMNFQGRLKKYLHGQNKKGKDGALLPPQLALFAIATPL---------   146
5NJ8_HsAhR_PASa     FLAMNFQGKLKKYLHGQKKKGKDGSILPPQLALFAIATPL---------   148
5Y7Y_HsAhRR_PASa    FLTMQFQGKLKFLFGQKKKAPSGAMLPPRLSLFCIAAPVL---------   155
5Y7Y_BtARNT_PASa    FVVVHCTGYIKAWPP--------------GSKFCLVAIGR---------   101
5Y7Y_BtARNT_PASb    WLWVRTSSFTFQNPYSDEIEYIICTNTNV--------------------   104
4ZP4_MmARNT_PASa    FVVVHCTGYIKAWPPAGVSLPDDDPEAGQGSKFCLVAIGRL--------   172
4ZP4_MmARNT_PASb    WLWMRTSSFTFQNPYSDEIEYIICTNTNV--------------------   103
1X0O_HsARNT_PASb    WLWMRTSSFTFQNPYSDEIEYIICTNTNVKNSSQE--------------   119
4F3L_MmBMAL1_PASB   FITLRSRWFSFMNPWTKEVEYIVSTNTVVL-------------------   106
4F3L_MmClock_PASb   WIWLQTHYYITYHQWNSRPEFIVCTHTVVSYAEVRAE------------   114
PA1181_PAS2         VRHLRAIATLTR-DADNRPVRMIGINSDITE------------------   116
PA4112_PAS3         IRFIQAGAQVER-DADGNPLQVTGINIDITSD-----------------   119
PA5442_PAS1         WRWLEDRGRAMARDPRGRVTRMLGTRSDISAR-----------------   119
PA0285_PAS1         YRWIHSRGRVLR-DALGKPLHYTGVARDITLQ-----------------   120
PA0338_PAS1         YLWISDRGRFVEFDERGEPRRMIGAHHEIHQR-----------------   121
1ZTU_DrBphp_PAS1    LLILEFEPTE---------------------------------------    93
5HSQ_AfAgp1_PAS     LVILEVEKA----------------------------------------    89
5AKP_XccBphP_nPAS   QWLVEMEPRDA--------------------------------------    96
3C2W_PaBphP_PAS1    VFYLEFEIRT---------------------------------------    90
PA4117_PAS1         VFYLEFEIRTADTLSITSF------------------------------   101
6G1Y_AfAgp2_PAS     TTIIEFEPSG---------------------------------------   100
4GW9_RpBphP1_PAS1   GLIIELERAG---------------------------------------    92
4GW9_RpBphP1_PAS2   DQSLGFVLIF---------------------------------------    97
2OOL_RpBphP3_PAS1   IVILELEPRDE--------------------------------------    97
4E04_RpBphP2_PAS    LVFLELEP-----------------------------------------    93
6PTQ_SaBphP2_PAS    LTVLELEPA----------------------------------------    85
2VEA_SsCph1_PAS1    LLVCELEPA----------------------------------------    97
6BAF_SaBphP_PAS     RLILELEPSS---------------------------------------    96
6JT0_HsSGCa_PAS     SRVMDLKGQMIYIVESSAILFLGSPC-----------------------    99
6JT0_HsSGCb_PAS     ISCLRLKGQMIYLPEADSILFLCSPS-----------------------   110
4GJ4_MsSGC_PAS      AEGLEIKGQMVFAAESDSLLFVGSPFL----------------------   101
1OJ5_MmNCoA-1_PAS1  MLSAHTRCKLCYPQSPDMQPFIMGIHIIDRE------------------   109
5NWM_HsNocoA1_PASb  MLSAHTRCKLCYPQSPDMQPFIMGIHIIDREHSGLSPQDDTNSGMSIPR   132
4HH2_RsPpsR_nPAS    RSILMLGRDL---------------------------------------    97
4HH2_RsPpsR_PAS1    LLLCQIDPA----------------------------------------    96
4HH2_RsPpsR_PAS2    RPLLVLVVRDTS-------------------------------------   100
4LRX_EcDhR_PAS      QF-IDAVITLKPIIETQ-----------GTSFILLLHPV----------    93
PA5124_PAS1         GVSITVDYAVTPILNRN-----------ETLLLLEVHPRDRL-------   114
5AKP_XccBphP_cPAS   GVTAHWLLQLR--------------------------------------   104
5LLW_ISPadC_PAS     YYIIEVERYH---------------------------------------    91
```

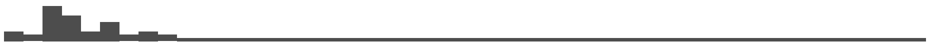

no cofactor or ligand binding

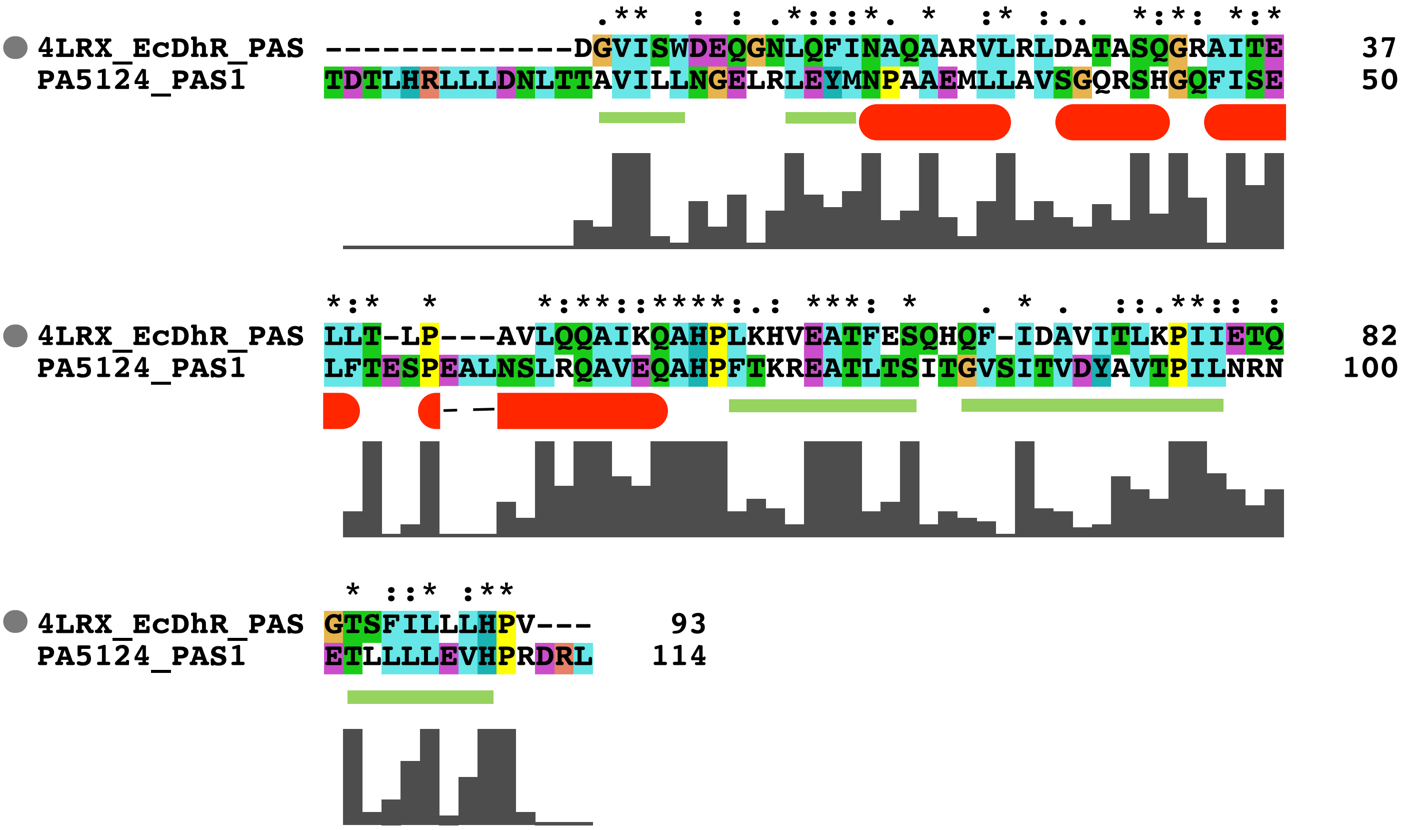
.** : : .*::::*. *   :* :..   *:*: *:*
4LRX_EcDhR_PAS -------------DGVISWDEQGNLQFINAQAARVLRLDATASQGRAITE 37
PA5124_PAS1 TDTLHRLLLDNLTTAVILLNGELRLEYMNPAAEMLLAVSGQRSHGQFISE 50
:   *   *:**::*****:.: ***: *   . * .   ::.**:: :
4LRX_EcDhR_PAS LLT-LP---AVLQQAIKQAHPLKHVEATFESQHQF-IDAVITLKPIIETQ 82
PA5124_PAS1 LFTESPEALNSLRQAVEQAHPFTKREATLTSITGVSITVDYAVTPILNRN 100
* ::* :**
4LRX_EcDhR_PAS GTSFILLLHPV--- 93
PA5124_PAS1 ETLLLLEVHPRDRL 114

Arrow 2

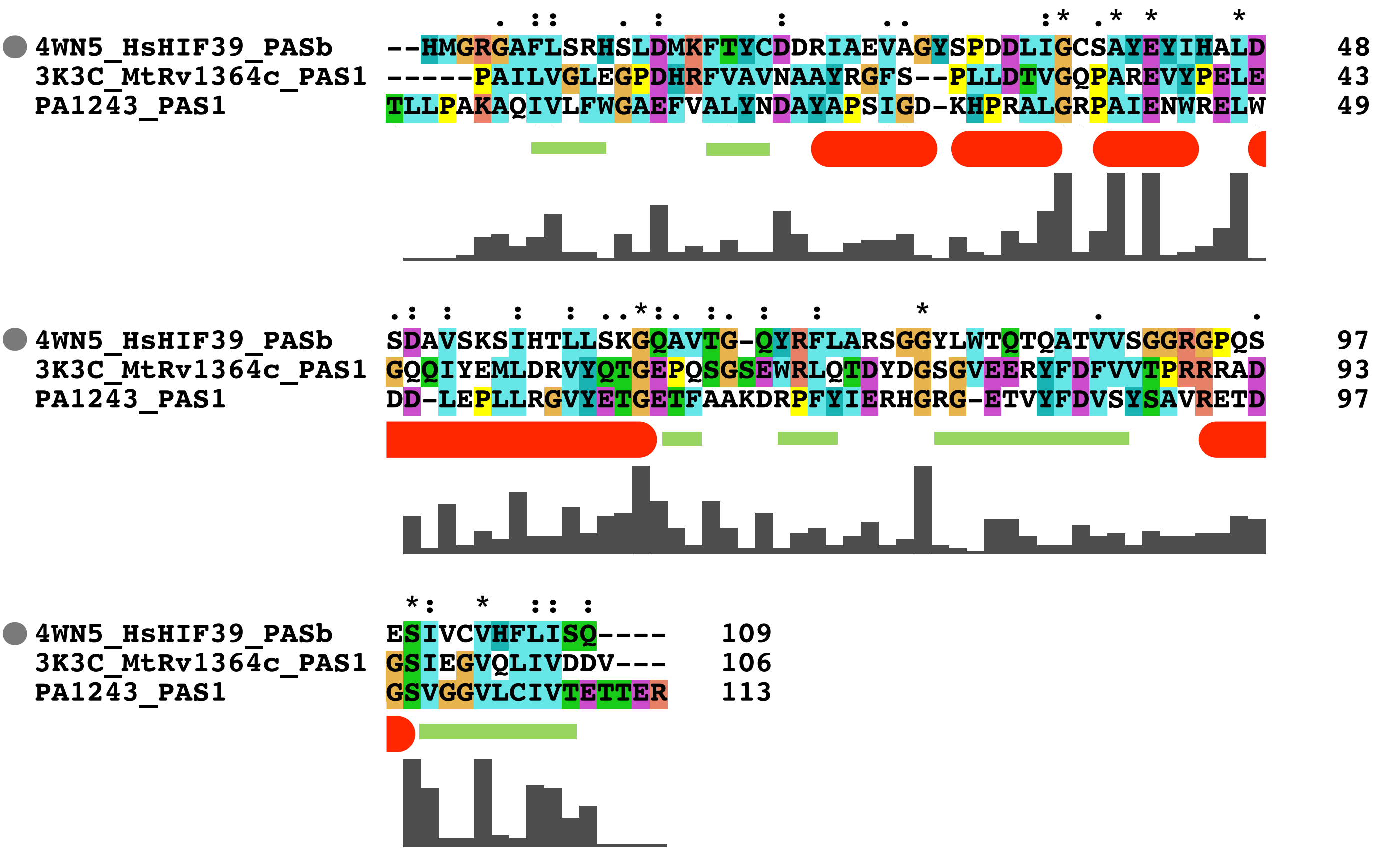
4WN5_HsHIF39_PASb --HMGRGAFLSRHSLDMKFTYCDDRIAEVAGYSPDDLIGCSAYEYIHALD 48
3K3C_MtRv1364c_PAS1 -----PAILVGLEGPDHRFVAVNAAYRGFS--PLLDTVGQPAREVYPELE 43
PA1243_PAS1 TLLPAKAQIVLFWGAEFVALYNDAYAPSIGD-KHPRALGRPAIENWRELW 49
4WN5_HsHIF39_PASb SDAVSKSIHTLLSKGQAVTG-QYRFLARSGGYLWTQTQATVVSGGRGPQS 97
3K3C_MtRv1364c_PAS1 GQQIYEMLDRVYQTGEPQSGSEWRLQTDYDGSGVEERYFDFVVTPRRRAD 93
PA1243_PAS1 DD-LEPLLRGVYETGETFAAKDRPFYIERHGRG-ETVYFDVSYSAVRETD 97
4WN5_HsHIF39_PASb ESIVCVHFLISQ---- 109
3K3C_MtRv1364c_PAS1 GSIEGVQLIVDDV--- 106
PA1243_PAS1 GSVGGVLCIVTETTER 113

Fatty acids

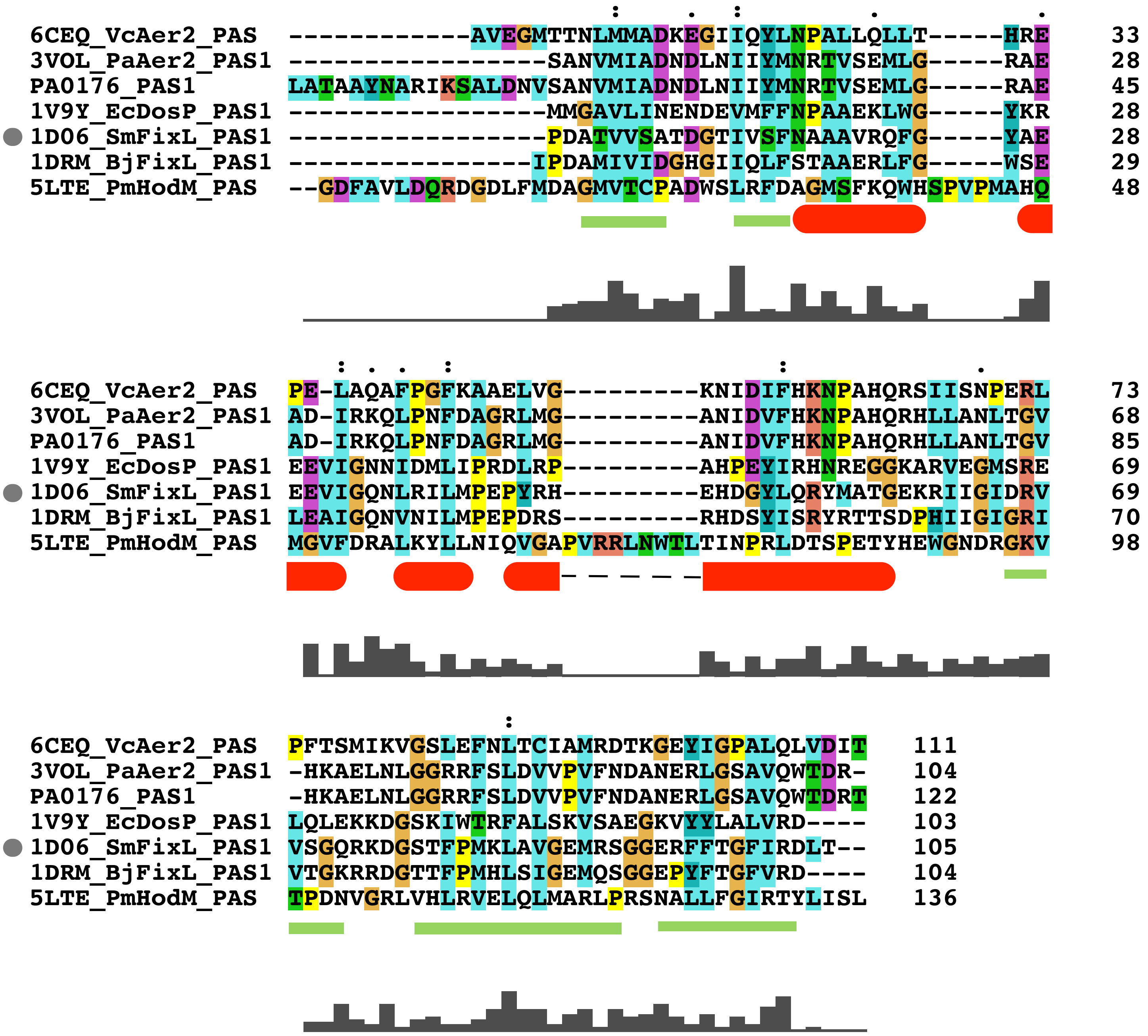

6CEQ_VcAer2_PAS ------------AVEGMTTNLMMADKEGIIQYLNPALLQLLT-----HRE 33
3VOL_PaAer2_PAS1 -----------------SANVMIADNDLNIIYMNRTVSEMLG-----RAE 28
PA0176_PAS1 LATAAYNARIKSALDNVSANVMIADNDLNIIYMNRTVSEMLG-----RAE 45
1V9Y_EcDosP_PAS1 -----------------MMGAVLINENDEVMFFNPAAEKLWG-----YKR 28
1D06_SmFixL_PAS1 -----------------PDATVVSATDGTIVSFNAAAVRQFG-----YAE 28
1DRM_BjFixL_PAS1 ----------------IPDAMIVIDGHGIIQLFSTAAERLFG-----WSE 29
5LTE_PmHodM_PAS --GDFAVLDQRDGDLFMDAGMVTCPADWSLRFDAGMSFKQWHSPVPMAHQ 48
6CEQ_VcAer2_PAS PE-LAQAFPGFKAAELVG---------KNIDIFHKNPAHQRSIISNPERL 73
3VOL_PaAer2_PAS1 AD-IRKQLPNFDAGRLMG---------ANIDVFHKNPAHQRHLLANLTGV 68
PA0176_PAS1 AD-IRKQLPNFDAGRLMG---------ANIDVFHKNPAHQRHLLANLTGV 85
1V9Y_EcDosP_PAS1 EEVIGNNIDMLIPRDLRP---------AHPEYIRHNREGGKARVEGMSRE 69
1D06_SmFixL_PAS1 EEVIGQNLRILMPEPYRH---------EHDGYLQRYMATGEKRIIGIDRV 69
1DRM_BjFixL_PAS1 LEAIGQNVNILMPEPDRS---------RHDSYISRYRTTSDPHIIGIGRI 70
5LTE_PmHodM_PAS MGVFDRALKYLLNIQVGAPVRRLNWTLTINPRLDTSPETYHEWGNDRGKV 98
6CEQ_VcAer2_PAS PFTSMIKVGSLEFNLTCIAMRDTKGEYIGPALQLVDIT 111
3VOL_PaAer2_PAS1 -HKAELNLGGRRFSLDVVPVFNDANERLGSAVQWTDR- 104
PA0176_PAS1 -HKAELNLGGRRFSLDVVPVFNDANERLGSAVQWTDRT 122
1V9Y_EcDosP_PAS1 LQLEKKDGSKIWTRFALSKVSAEGKVYYLALVRD---- 103
1D06_SmFixL_PAS1 VSGQRKDGSTFPMKLAVGEMRSGGERFFTGFIRDLT-- 105
1DRM_BjFixL_PAS1 VTGKRRDGTTFPMHLSIGEMQSGGEPYFTGFVRD---- 104
5LTE_PmHodM_PAS TPDNVGRLVHLRVELQLMARLPRSNALLFGIRTYLISL 136

Heme b

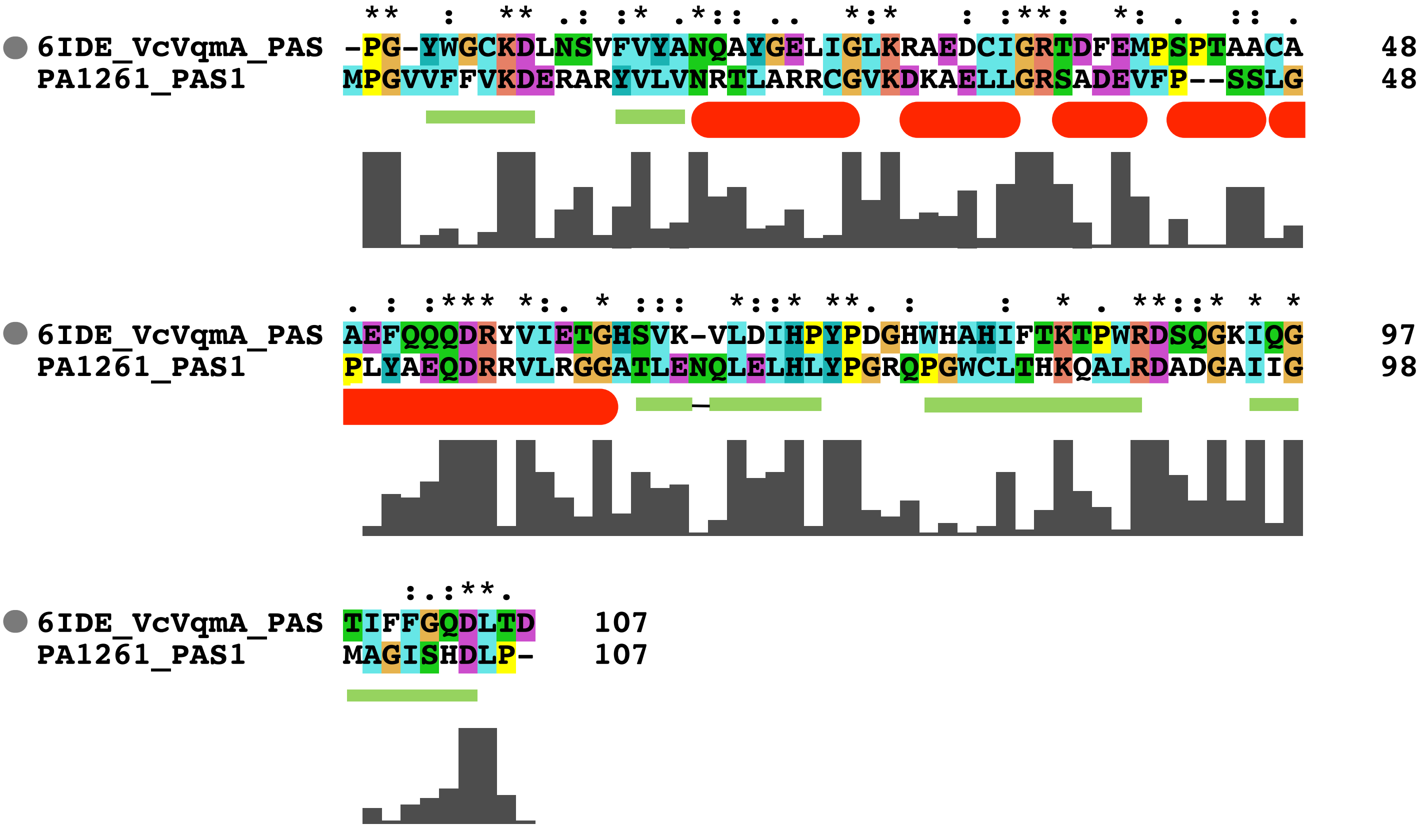

Autoinducers

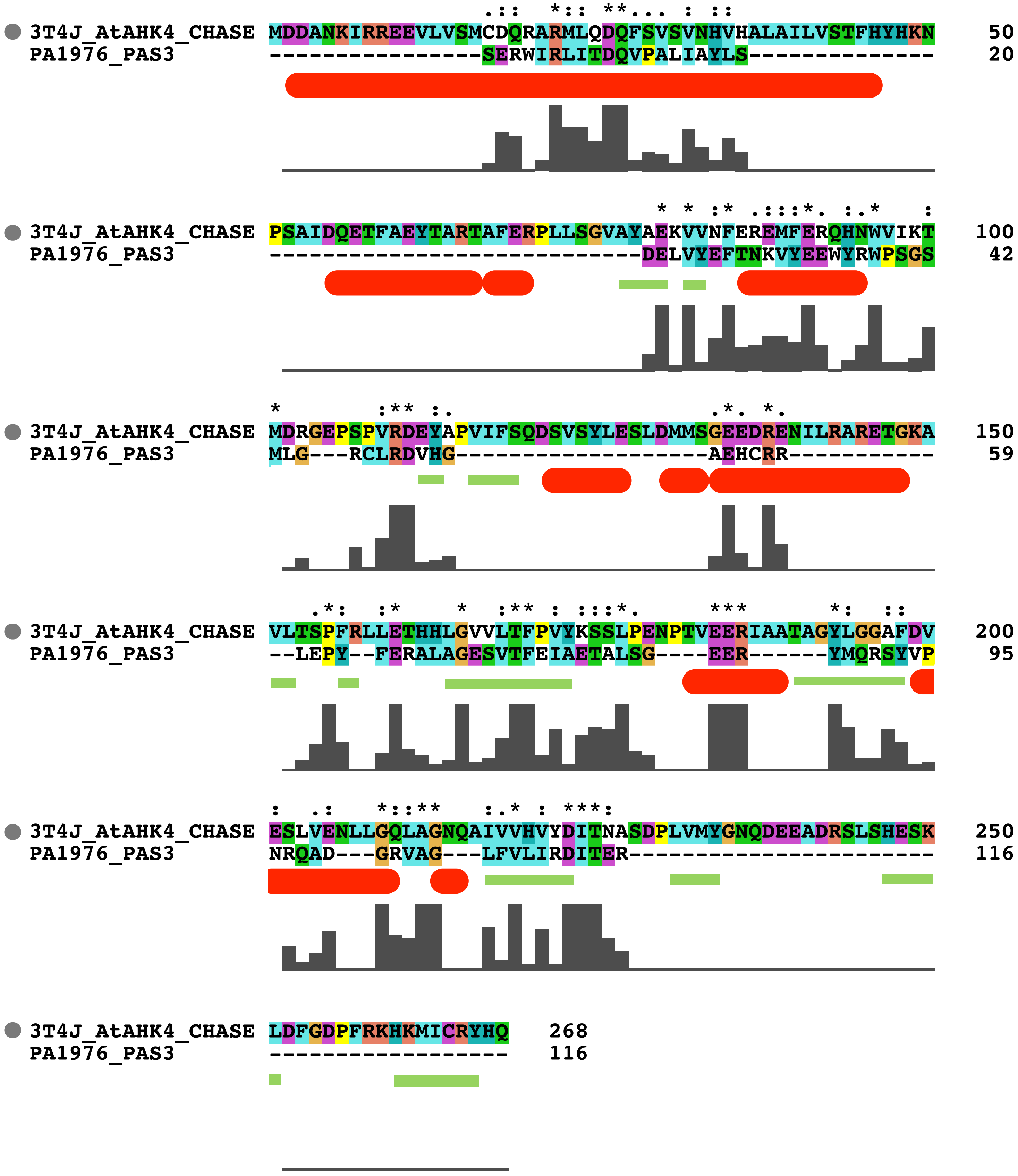
.::   *:: **... : ::
3T4J_AtAHK4_CHASE MDDANKIRREEVLVSMCDQRARMLQDQFSVSVNHVHALAILVSTFHYHKN 50
PA1976_PAS3 ----------------SERWIRLITDQVPALIAYLS-------------- 20
* * :* .:::*. :.*   :
3T4J_AtAHK4_CHASE PSAIDQETFAEYTARTAFERPLLSGVAYAEKVVNFEREMFERQHNWVIKT 100
PA1976_PAS3 ----------------------------DELVYEFTNKVYEEWYRWPSGS 42
*   :** :.   .*. *.
3T4J_AtAHK4_CHASE MDRGEPSPVRDEYAPVIFSQDSVSYLESLDMMSGEEDRENILRARETGKA 150
PA1976_PAS3 MLG---RCLRDVHG-------------------AEHCRR----------- 59
.*:   :*   *   :** : ::::*.   ***   *:   ::
3T4J_AtAHK4_CHASE VLTSPFRLLETHHLGVVLTFPVYKSSLPENPTVEERIAATAGYLGGAFDV 200
PA1976_PAS3 --LEPY--FERALAGESVTFEIAETALSG----EER------YMQRSYVP 95
:   .:   *::**   :.* : ***:
3T4J_AtAHK4_CHASE ESLVENLLGQLAGNQAIVVHVYDITNASDPLVMYGNQDEEADRSLSHESK 250
PA1976_PAS3 NRQAD---GRVAG---LFVLIRDITER----------------------- 116
3T4J_AtAHK4_CHASE LDFGDPFRKHKMICRYHQ 268
PA1976_PAS3 ------------------ 116

Cytokinins

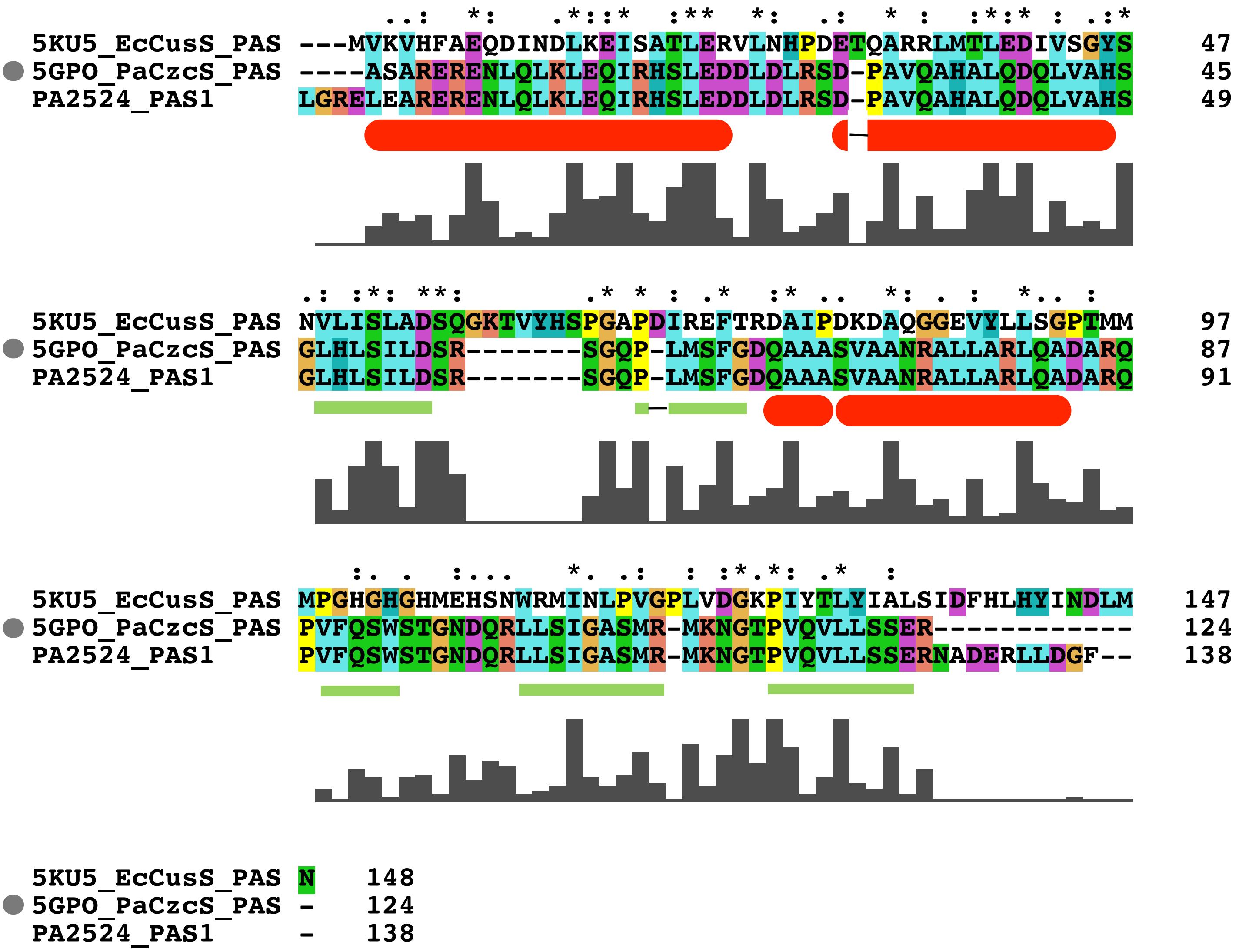
5KU5_EcCusS_PAS ---MVKVHFAEQDINDLKEISATLERVLNHPDETQARRLMTLEDIVSGYS 47
5GPO_PaCzcS_PAS ----ASARERENLQLKLEQIRHSLEDDLDLRSD-PAVQAHALQDQLVAHS 45
PA2524_PAS1 LGRELEARERENLQLKLEQIRHSLEDDLDLRSD-PAVQAHALQDQLVAHS 49
5KU5_EcCusS_PAS NVLISLADSQGKTVYHSPGAPDIREFTRDAIPDKDAQGGEVYLLSGPTMM 97
5GPO_PaCzcS_PAS GLHLSILDSR-------SGQP-LMSFGDQAAASVAANRALLARLQADARQ 87
PA2524_PAS1 GLHLSILDSR-------SGQP-LMSFGDQAAASVAANRALLARLQADARQ 91
5KU5_EcCusS_PAS MPGHGHGHMEHSNWRMINLPVGPLVDGKPIYTLYIALSIDFHLHYINDLM 147
5GPO_PaCzcS_PAS PVFQSWSTGNDQRLLSIGASMR-MKNGTPVQVLLSSER------------ 124
PA2524_PAS1 PVFQSWSTGNDQRLLSIGASMR-MKNGTPVQVLLSSERNADERLLDGF-- 138
5KU5_EcCusS_PAS N 148
5GPO_PaCzcS_PAS - 124
PA2524_PAS1 - 138

Metals (PAS)
